# Inferring the qualities of protein–RNA models with graph transformers

**DOI:** 10.1093/bioinformatics/btag202

**Published:** 2026-04-28

**Authors:** Andrew Jordan Siciliano, Yifan Bao, Bishal Shrestha, Zheng Wang

**Affiliations:** Department of Computer Science, University of Miami, Coral Gables, FL 33124, United States; Department of Computer Science, University of Miami, Coral Gables, FL 33124, United States; Department of Computer Science, University of Miami, Coral Gables, FL 33124, United States; Department of Computer Science, University of Miami, Coral Gables, FL 33124, United States

## Abstract

**Motivation:**

Breakthrough advancements in protein tertiary and quaternary structure prediction have accelerated structural bioinformatics research activity and drug development processes. However, many biological mechanisms involve more complicated interactions, such as those between amino and nucleic acids. Predicting the structure of protein–RNA complexes is highly relevant and challenging due to data scarcity and experimental difficulties. Understanding and interpreting these interactions can yield crucial insights into various human diseases and biological phenomena. Thus, quality assessment methods that specifically evaluate protein–RNA complex models can provide significant utility in this emerging area of protein–RNA structural bioinformatics research.

**Results:**

We propose a novel graph transformer-based approach named complex quality assessment of RNA and protein (CARP) to infer multiple quality perspectives of protein–RNA complex models. For a single protein–RNA complex model, in one shot, CARP simultaneously predicts multiple overall fold, overall interface, and per-protein–RNA interface quality estimates. When evaluated against a non-redundant protein–RNA docking benchmark, our methods demonstrated obvious improved performance compared to almost all of the existing scoring tools, particularly when ordering and selecting the highest quality decoys. Furthermore, CARP consistently selected higher quality models relative to other predictors when tested on CASP16 targets. Specifically, CARP-predicted global interface and global protein–RNA interface qualities were ranked first and second, respectively, based on the selected top-3 models over all ten CASP16 protein–RNA complex targets. CARP also showed a strong ability, compared to both existing tools and AlphaFold3 self-estimates, in selecting high quality AlphaFold3 models.

**Availability and implementation:**

CARP is freely available at github.com/zwang-bioinformatics/CARP/.

## 1 Introduction

Protein–RNA complexes are involved in many fundamental biological processes (Glisovic et al. 2008, Chung et al. 2019, Ramanathan et al. 2019). Structural abnormalities in RNA–protein interactions can lead to various human diseases, such as AIDS and cancer (Khalil and Rinn 2011, Ning et al. 2021). Understanding the structure of these complexes can provide vital insights into these diseases and help investigate targeted therapies. While current experimental tools, such as X-ray crystallography, NMR, and Cryo-EM, have provided direct access to the 3D structures of protein–RNA complexes, they are expensive and time-consuming, especially for protein–RNA complexes (Ke and Doudna 2004, Scott and Hennig 2008). Thus, computational tools that accurately predict protein–RNA complex structures can significantly advance the study of their interactions.

Methods to predict protein–RNA complex structures typically involve either expert modeling knowledge, deep learning techniques, or computational docking protocols. While some methods are occasionally successful, many approaches to protein–RNA structure prediction are not nearly as accurate as those for protein tertiary and quaternary structure prediction tasks (Kretsch et al. 2026). Thus, computational approaches for scoring and ranking protein–RNA structural models can help prioritize the most accurate predictions for further usage of the models.

Statistical potential-based scoring functions are a common approach to score protein–RNA interactions (Qiu and Zou 2020). For example, DARS-RNP (Tuszynska and Bujnicki 2011), QUASI-RNP (Tuszynska and Bujnicki 2011), FTDMP (Olechnovič et al. 2025), 3dRPC-Score (Li et al. 2017), and ITScore-PR (Huang and Zou 2014) all use statistical potentials to score protein–RNA structural decoys. These focus either on coarse-grained or atomic-level representations of the structures. Coarse-grained scoring functions are less sensitive to minor structural alterations and benefit from improved computational complexity. A significant limitation of coarse-grained scoring functions is their inability to examine detailed atomic-level molecular interactions. Atomic-level scoring functions analyze structures in high resolution, enabling them to achieve high accuracy when scoring structures close to nature. However, atomic-level scoring functions struggle with increased computational complexity and can be more sensitive to complex conformational changes, especially for flexible RNA and protein chains (Zeng et al. 2024).

Deep learning methods have been developed to overcome the limitations of knowledge-based scoring functions. For example, DRPScore (Zeng et al. 2023) is a recently developed deep-learning-based protein–RNA complex scoring function, which uses a 4D convolutional neural network (4DCNN) to evaluate protein–RNA complex interfaces. Furthermore, in the context of protein-complex quality assessment, Graph neural networks (GNNs) have proven effective in inferring the quaternary structural qualities of proteins (Olechnovič and Venclovas 2023, Réau et al. 2023).

We propose a novel graph-transformer-based method named CARP for inferring the quality of individual protein–RNA complex models. The topology of an input structure is embedded within a graph by encoding residues as nodes and spatial interactions between residues as edges. This graph is then processed by a state-of-the-art deep-learning pipeline composed of graph-transformer layers (Shi et al. 2021, Rampášek et al. 2022) and global-attention-pooling layers (Li et al. 2016) for quality assessment.

## 2 Materials and methods

### 2.1 Datasets

We first obtained DRPScore’s (Zeng et al. 2023) training set and cross-referenced them with both the CASP16 targets (used as blind-test) and DRPScore’s (Zeng et al. 2023) blind-test dataset (DRP-Testing Set 2 docking benchmark). We then filtered all protein–RNA complexes in the training set that were similar to the blind test complexes. Specifically, we removed any complex that had, for any chain, a protein sequence similarity of ≥0.3, or an RNA sequence similarity of ≥0.7, using MMseqs2 (Steinegger and Söding 2017), or any overlapped RFam (Ontiveros-Palacios et al. 2025) clans to any RNA sequence in the blind tests. Furthermore, we used USAlign (Zhang et al. 2022) to detect structural similarities between the training set and the blind test sets, using a TM-score threshold of 0.5, except for the CASP16 target M1216, which has a TM-score of ≈0.6 with the PDB ID 6VRD in our training dataset (due to restricted CASP targets). This comprehensive filtering process ensures the dataset’s quality and non-redundancy, resulting in a final training set of 150 protein–RNA complexes.

To partition our training set into validation splits, we used a stratified 5-fold approach. Specifically, we clustered the training complexes using MMseqs2 (Steinegger and Söding 2017) with a protein-sequence similarity cutoff of 0.6. We also defined clusters using RFam (Ontiveros-Palacios et al. 2025), yielding two definitions: one based on protein-sequence similarity and the other on RNA-sequence clan classification. Given the highly imbalanced cluster distribution, characterized by a few massive clusters and numerous singletons, we adopted a stratified sampling strategy to ensure representative fold construction. For each RFam clan, we randomly distributed its complexes across the five folds, ensuring each fold was representative. The remaining complexes, to which no RFam clan was assigned, were then distributed similarly, except based upon the protein-sequence clusters. This approach was carefully designed to produce a reliable and balanced set of five folds, where each served as a validation set for an ensemble of five graph transformer models, more details can be found in Section 2.3.

To generate the protein–RNA structural decoys for training, we utilized docking protocols and structural perturbation techniques. Similar to (Zeng et al. 2023), we executed 3dRPC (Li et al. 2017) to generate 10k decoys for each native structure, see Supplementary 1.2, available as supplementary data at *Bioinformatics* online. We then selected the 500 decoys closest to the native structure, in terms of RMSD, to be included in our training set. In addition to these 500 decoys, we used PyRosetta (Chaudhury et al. 2010) to further perturb both the top-100 (closest to the native structure) of the 3dRPC generated decoys and the original native structure of the complex, making an average of ≈2.7k decoys for each of the 150 complexes in the training set.

When we used PyRosetta to perturb proteins for training, we utilized small and shear movers. When we used PyRosetta to perturb RNA structures for training, we utilized fragment movers, which randomly insert fragments sampled from a database of RNA fragments and respective torsion angles (Leontis and Zirbel 2012, Watkins et al. 2020). Our overall dataset (all five folds) contained, in total, ≈400k decoys (a combination of all decoys across all complexes). We used the tool OpenStructure (Biasini et al. 2013) to calculate the three global folding, three global interface, and two per-interface ground truth quality scores, see Table 2, available as supplementary data at *Bioinformatics* online.

To test our CARP and six other state-of-the-art tools, we evaluated them against the blind test protein–RNA complexes provided by DRPScore (Zeng et al. 2023) (DRP-Testing Set 2 docking benchmark). We generated two blind-test sets from the DRPScore testing set 2: the first was a docking benchmark, where we used 3dRPC (Li et al. 2017) to generate the 1k decoys per complex, and the second test set comprised 100 AlphaFold3 (Abramson et al. 2024) (AF3) models per complex (20 seeds, each with 5 diffusion samples). Ground truth scores on these two test sets were computed using a custom script, built upon OpenStructure (Biasini et al. 2013), that can calculate iRMSD specifically for protein–RNA evaluation, see Supplementary 1.6, available as supplementary data at *Bioinformatics* online. We also filtered, for each tool, the blind-test complexes that were similar to each respective training set. Further details can be found in the Supplementary 2.2, available as supplementary data at *Bioinformatics* online.

To assess performance on real-world modeling tasks, we further evaluated our CARP and the other six state-of-the-art tools based on the ten CASP16 (Kretsch et al. 2026) protein–RNA targets. The true quality scores for CASP16 models were either directly obtained from the CASP16 website or computed by our custom script (depending on the native structure’s availability). We ignored the CASP16 models without ground-truth scores and only kept the CASP16 models that contain both protein and RNA residues, making a total of ten targets.

### 2.2 Graph topologies and feature generation

Our deep learning pipeline takes as input a graph-encoded protein–RNA complex model. Each node represents either an amino acid or a nucleotide, for protein and RNA chains, respectively. Edges encode spatial interactions between residues in the complex. An edge is defined between a pair of residues if their anchor atoms’ (defined later) distance is ≤14 Å or any atom-atom distance is ≤6 Å. Anchor atoms are defined as Cβ (Cα for Glycine) for protein residues and C3’ for RNA residues.

Node features are comprised of 66 channels and include both predicted (e.g. prediction from sequence) and annotated (e.g. parsed from the model) features. For the proteins, we executed DSSP (Kabsch and Sander 1983) and NetSurfP-3.0 (Høie et al. 2022) to generate the annotated and predicted features, respectively, in terms of secondary structure, relative surface accessibility, and torsion angles. RNA residue annotations include torsion angles (η and θ) from AMIGOS (Duarte and Pyle 1998) and interacting nucleotides from RNAView (Yang et al. 2003) and Forgi (Thiel et al. 2019). We also included a binary indicator for whether the residue belongs to a protein or RNA chain and whether the residue is part of a protein–RNA interface. Interface residues between protein–RNA chains are defined as the residues that have any atom-atom distance between the residue and opposing chain ≤6 Å. We include the distance and angle from each residue to the center of mass of the entire complex and of the residue’s respective chain (can be a protein or RNA chain), as well as a one-hot encoding of the amino acid and nucleotide types.

Edge features contain 12 channels and include both predicted and annotated features. Four channels are dedicated to model-calculated distances (atom-atom and anchor-anchor) and angles between the residues (can both be protein or RNA residues, or one protein and one RNA residue). We included predicted angles and distances for intra-protein interactions from AlphaFold2 (Jumper et al. 2021, Mirdita et al. 2022). Annotated base-pair interactions between RNA residues were generated using RNAView, and predicted base-pair interactions between RNA residues were obtained by executing LinearPartition (Zhang et al. 2020) and IPKnot (Sato et al. 2011). We included two binary indicators for whether the interaction is inter-chain and whether the interaction occurs between a protein and an RNA chain. Full details of node and edge features are shown in Table 1, available as supplementary data at *Bioinformatics* online.

### 2.3 Deep learning architecture

Our deep learning model was trained to predict, from a single input protein–RNA complex graph, three global fold scores, three global interface scores, and two scores for each protein–RNA interface, simultaneously. The three global fold scores are denoted as ${\hat{F}}_{0},\;{\hat{F}}_{1},\;\text{and}\;{\hat{F}}_{2}$, corresponding to the predicted values of bb-LDDT, Oligo-GDTTS, and Oligo-GDTHA, respectively. We denote the three global interface scores as ${\hat{I}}_{0},\;{\hat{I}}_{1},\;\text{and}\;{\hat{I}}_{2}$, corresponding to the predicted values for iLDDT, IPS, and ICS, respectively. The two scores per protein–RNA interface are defined as ${\hat{P}}_{0}[i]\;\text{and}\;{\hat{P}}_{1}[i]$, corresponding to predicted values for the per-interface IPS and ICS for the *i*th protein–RNA interface, respectively. In total, for a single input graph, our deep learning model simultaneously predicts six global quality scores (for the entire graph) and two quality scores for each protein–RNA interface (subgraphs).

Our pipeline consists of three components, each followed by a pooling layer that learns to drop less relevant nodes via top-k pooling (Gao and Ji 2019). The first and only layer that processes all nodes at once (before dropping any node) is the GPSConv (Rampášek et al. 2022), which is intentional, as GPSConv utilizes multihead attention (Vaswani et al. 2017), i.e., information is mixed from every node to every other node. Note the distances and angles to the center of mass encode the relative locations for amino acid or nucleotide residues at the node level.

In other words, the deep learning pipeline first processes the input graph, $G_{0}$, using GPSConv, which applies multihead attention (Vaswani et al. 2017) over the node features, and GINEConv (Hu et al. 2020), which applies a single linear layer over each node and its aggregated neighborhood, to produce an updated graph $G_{1}$. This updated graph $G_{1}$ is then processed by the second component, a TransformerConv (Shi et al. 2021) layer, to produce $G_{2}$. This second component is included to allow information propagation in a traditional message-passing way while still incorporating attention mechanisms for improved expressibility. Following this, we introduced inductive biases for the protein–RNA interface interactions in the algorithm. To do this, we removed the edges in $G_{2}$ that are not connecting protein and RNA residues and pass the pruned ${\tilde{G}}_{2}$ to the third component, a TransformerConv (Shi et al. 2021), which outputs $G_{3}$.

**Figure 1:**
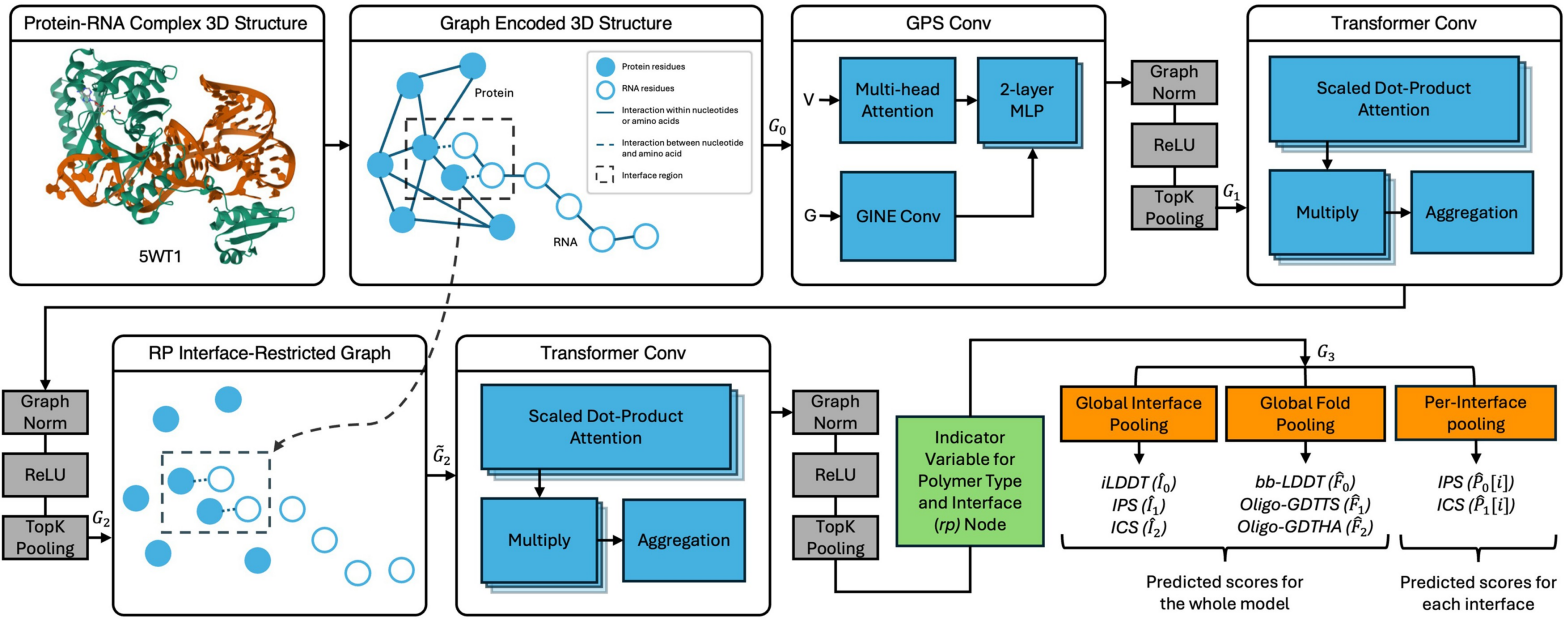
Deep learning architecture of CARP.

To produce the global fold $\left({\hat{F}}_{0},\;{\hat{F}}_{1},\;\text{and}\;{\hat{F}}_{2}\right)$, overall interface $\left({\hat{I}}_{0},\;{\hat{I}}_{1},\;\text{and}\;{\hat{I}}_{2}\right)$, and per-interface $\left({\hat{P}}_{0}[i]\;\text{and}\;{\hat{P}}_{1}[i]\right)$ qualities, we use three global attention pooling (GAP) (Li et al. 2016) layers. These layers act independently on the learned node features of $G_{3}$, concatenated with the indicator variables for protein–RNA interface and polymer type (protein or RNA), to help the algorithm better identify which residues are important for different scores. Note that for all three global attention pooling layers, the same per-node feature representation is used as input, meaning these learned features are guided to learn a local representation that can provide information for all quality metric types. The Sigmoid function is applied to all outputs to produce the final predicted qualities. Top-k pooling (Gao and Ji 2019), GraphNorm (Cai et al. 2021), and ReLU activation are utilized throughout the pipeline. For more details, see Fig. 1 and Algorithm 1, available as supplementary data at *Bioinformatics* online.

For each fold in the five folds, we performed hyper-parameter tuning and picked the best model based on validation loss. We then averaged the predictions of these configurations to produce the output predictions for downstream evaluations. The learning curves for these five best-performing architectures and results for hyper-parameter tuning can be found in Supplementary 1.5, available as supplementary data at *Bioinformatics* online, and in the Supplementary Data.

Our deep learning model predicts six global, e.g., bb-LDDT, iLDDT, and ICS, and two per-interface quality scores, e.g., IPS and ICS, which are then combined in different ways to build four global and two per-interface scores as the final outputs from our pipeline. Our loss function, detailed in Supplementary 1.4, available as supplementary data at *Bioinformatics* online, is composed of averaged L1 (absolute difference) losses for each ground-truth score. Therefore, averaging was also used to produce the combined predicted scores from CARP. Specifically, to produce the global fold prediction, or CARP-Fold, we average the three global fold predictions ($\hat{F}$), i.e., predicted bb-LDDT, Oligo-GDDTS, and Oligo-GDTHA (see Equation 1, available as supplementary data at *Bioinformatics* online). To produce the global interface prediction, referred to as the CARP-Iface score, we average the three global interface predictions ($\hat{I}$), i.e., predicted iLDDT, IPS, and ICS (see Equation 2, available as supplementary data at *Bioinformatics* online). We also define a score that covers both the global fold and interface qualities, denoted as the CARP-Merged score, which is the average of the global fold and global interface predicted scores (see Equation 3, available as supplementary data at *Bioinformatics* online). For interface *i*, the per-protein–RNA interface quality, or the CARP-PerIF score, is defined as the average of the two per-protein–RNA-interface scores ($\hat{P}[i]$), i.e., predicted interface level ICS and IPS (see Equation 4, available as supplementary data at *Bioinformatics* online). We define a per-interface score, referred to as the CARP-PerIF-G score, that also incorporates information about the global interface, by averaging, for each interface, the per-protein–RNA interface quality (PerIF) and the global interface quality scores (see Equation 5, available as supplementary data at *Bioinformatics* online). The global protein–RNA interface quality, or the CARP-RP score, is defined as the average of all of the predicted per-protein–RNA interface scores ($\hat{P}$) (see Equation 6, available as supplementary data at *Bioinformatics* online). Further information regarding the ground truth scores can be found in Supplementary 1.6, available as supplementary data at *Bioinformatics* online.

## 3 Results and discussion

We compared our tool against six state-of-the-art tools for evaluating protein–RNA docking poses and model qualities. Table 3, available as supplementary data at *Bioinformatics* online, details the type of scores predicted by our tool and the comparison tools for global and per-interface qualities. Note that we negate energy (*E*) scores to standardize the direction of the quality scores (higher values indicate better quality and vice versa). All methods for comparison, including our CARP, are treated as single-model quality predictors.

We computed two performance measures to evaluate predictors on the CASP16 benchmark. The first metric we utilized is Ranking Loss (RL). For an ensemble of models with quality scores *M* and predicted qualities $\hat{M}$, Ranking Loss (RL) (Studer et al. 2023) is defined as the deviation in the ground truth score of a predictor’s top-ranked model and the true top-ranked model, see Equation (1).


(1) $$\text{RL}(M,\hat{M})=|\text{best}(M)-M[\mathrm{argmax}(\hat{M})]|$$

The second performance metric that we utilized was the per-target CASP16 model Z-score, which was used by the CASP16 official assessment (Kretsch et al. 2026), see Equation (2). For each CASP16 target, we computed Z-scores over all of the structural models predicted by CASP16 participating groups, referred to as Z-CASP16. The mean and standard deviation for the Z-score calculations excluded low-quality outliers (Kretsch et al. 2026), i.e., individual Z-score <−2.


(2) $$\begin{array}{cc} \text{Z-CASP16}=0.3(0.3Z_{\text{TM}}+0.3Z_{\text{GDT}\_\text{TS}}+0.4Z_{\text{LDDT}}) & \\ +0.7(\frac{1}{3}Z_{\text{ICS}}+\frac{1}{3}Z_{\text{IPS}}+\frac{1}{3}Z_{\text{iLDDT}}) & \end{array}$$

**Figure 2:**
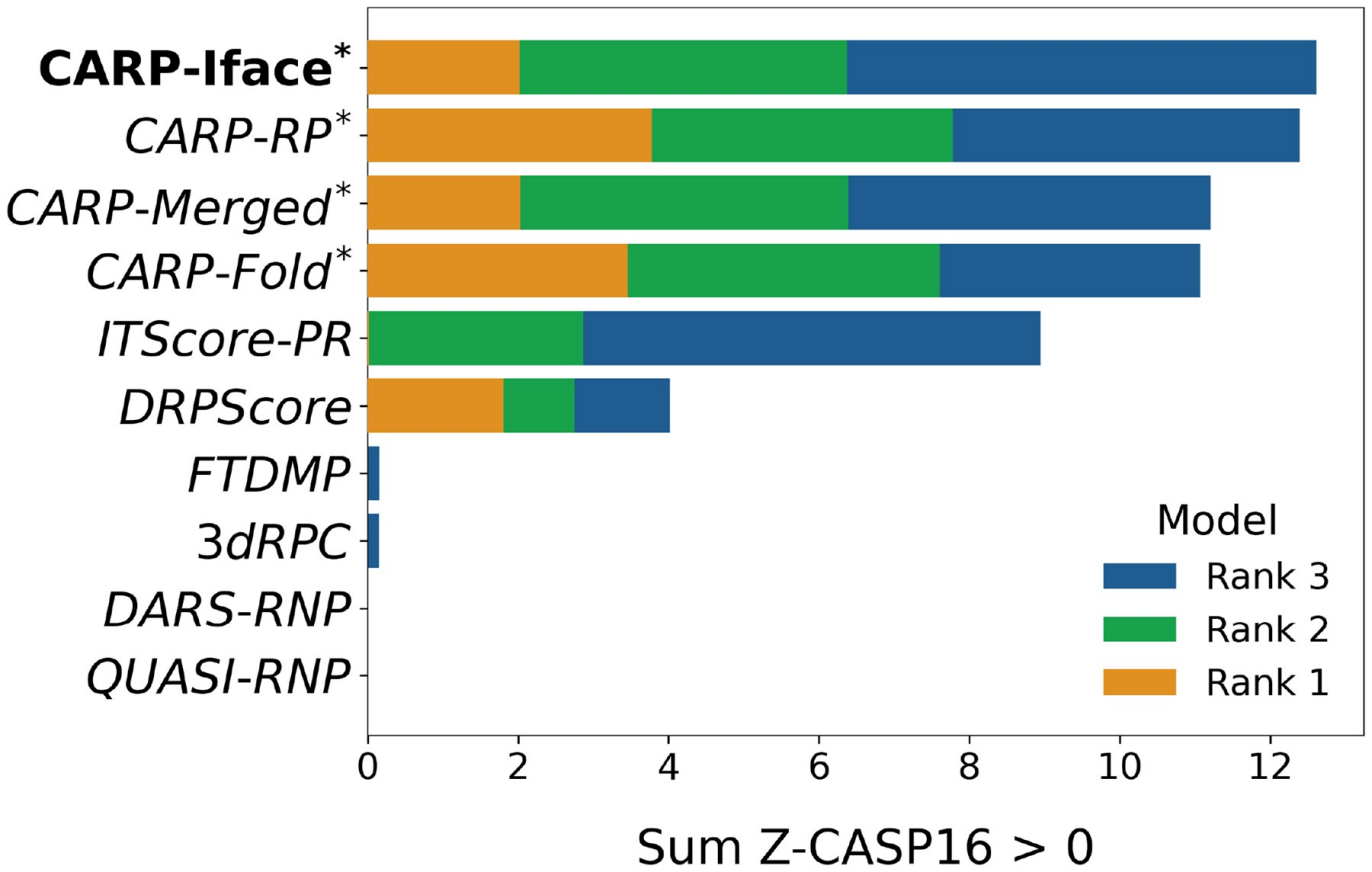
Quality predictor rankings for CASP16 protein–RNA complex targets, in terms of the sum of the three top-selected model’s Z-CASP16 scores, over all 10 targets. Bold indicates the best method, and (*) indicates one of our methods (CARP).

Similar to how CASP16 evaluated the protein–RNA structural predictors, we ranked the quality predictors in terms of the sum of the Z-CASP16 scores (with negative Z-CASP16 scores set to zero) of the top-3 models (ranked based on the predicted quality scores). These summed scores directly indicate the overall performance for ranking the top-3 highest quality models. The rankings and summed Z-CASP16 scores are presented in Fig. 2, where CARP-Iface, CARP-RP, CARP-Merged, and CARP-Fold predicted qualities ranked first, second, third, and fourth, respectively. These results indicate that CARP outperforms the other six state-of-the-art methods in selecting the most accurate protein–RNA complex models.

To further assess the ability of the predictors for selecting the best model, we plotted Fig. 6, available as supplementary data at *Bioinformatics* online, that shows, for each CASP16 target, the RMSD Ranking Loss and Z-CASP16 of all predictors’ top-ranked models, which further solidify our findings from Fig. 2. Across the majority of the ten targets, CARP, specifically CARP-Fold, stays in the upper range of predictors for both metrics.

**Figure 3:**
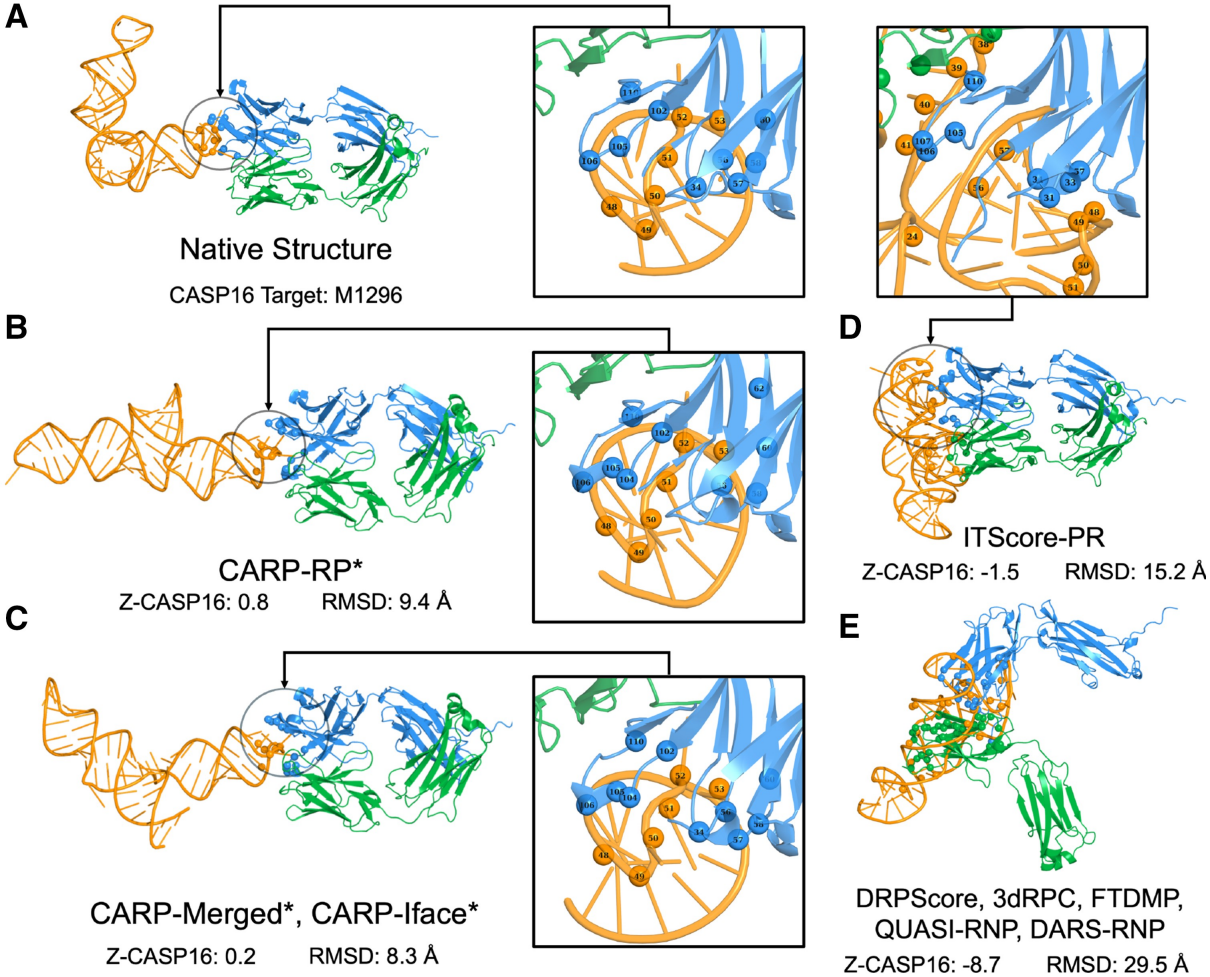
(A) The structure and protein–RNA interface contacts for the native CASP16 target M1296, (B) the top model for CARP-RP, (C) CARP-Merged and CARP-Iface, (D) ITScore-PR, and (E) for all other tools. Structures were rendered using PyMol (Schrödinger 2015) and residues involved in protein–RNA interface (polar) contacts are shown as spheres.

Figure 3 shows the native structure of CASP16 target M1296, and the top model selected by each tool. We also show an enlarged view of the residues involved in protein–RNA interface (polar) contacts. For this target, it is evident that CARP is the superior tool for selecting higher quality models. Specifically, the CARP selected models better resemble the overall fold configuration and also better capture the intricate local protein–RNA interface contacts.

**Figure 4:**
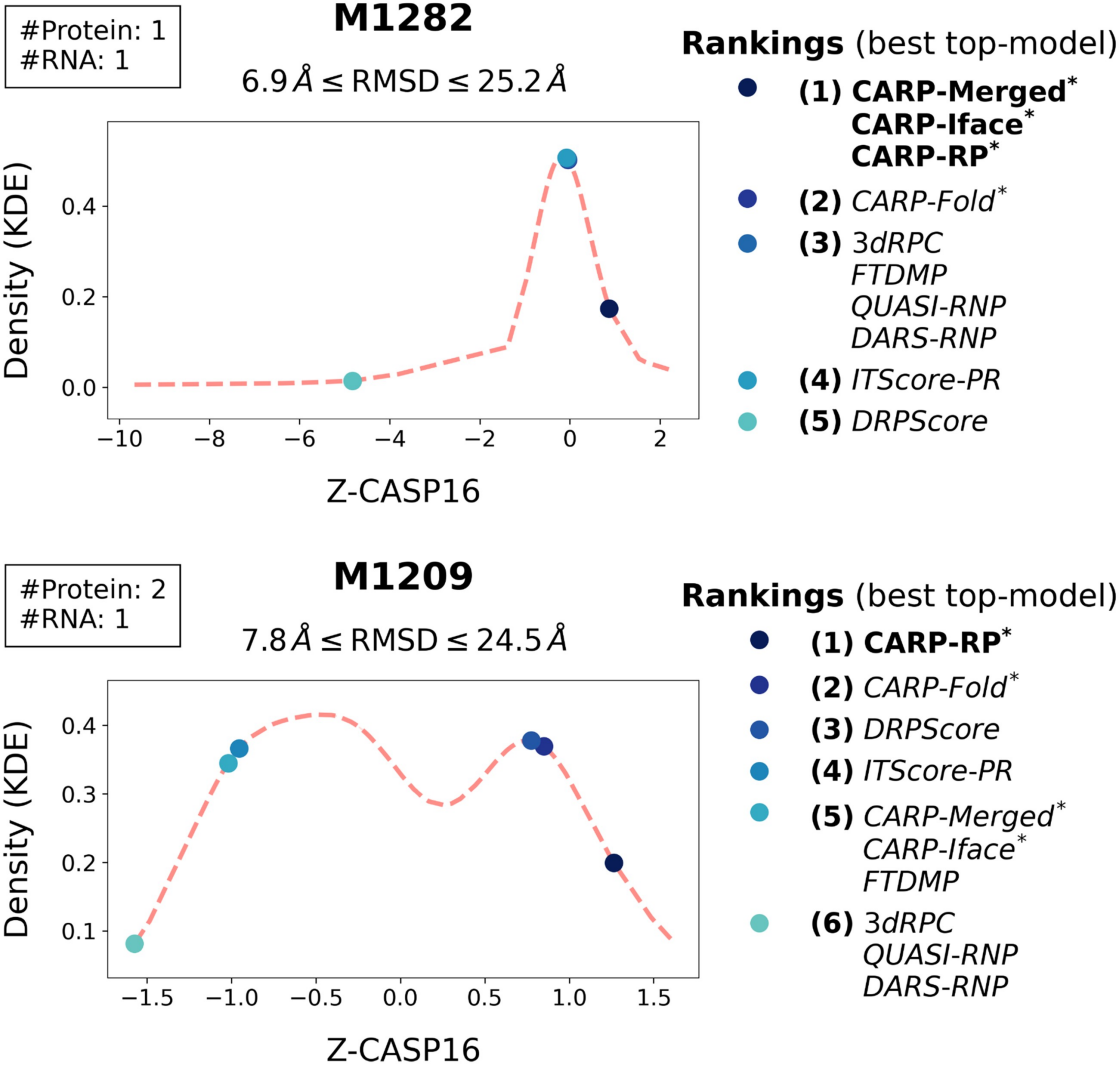
Quality predictor rankings for two CASP16 protein–RNA complex targets, M1282 and M1209, in terms of the selected top-model’s Z-CASP16 score. The dashed line indicates the probability density estimation (*y*-axis) of a model with a particular Z-CASP16 score (*x*-axis). Methods in bold selected the best model relative to other predictors and (*) indicates our CARP.

The performance of structure modeling groups during CASP16 indicated that the target M1209 was easier to model and the target M1282 was more difficult to model (Kretsch et al. 2026). In both cases, we see CARP quality estimates select better quality models comparatively to the other scoring tools. Figure 4 shows, for both targets, the true rankings of top-selected models along with the corresponding probability density estimation (*y*-axis) of a model with that particular Z-CASP16 score (*x*-axis). Even for the difficult-to-model M1282, we find that all four of the combined predicted global qualities by CARP outperform the other evaluated scoring methods. Notably, CARP not only selected higher-quality models but also selected models with low probability densities. In other words, these low-probability-density models are hard to be chosen by chance, but CARP achieved that.

Figure 10, available as supplementary data at *Bioinformatics* online, shows for all targets the density (KDE) and location of the best-ranked model for each method in terms of Z-CASP16. For many of the targets, CARP excels at finding higher quality models compared to other scoring functions. While modeling protein–RNA complexes is still a computationally challenging endeavor, as indicated by the CASP16 results and subsequent analysis (Kretsch et al. 2026), our results demonstrate that CARP can be a valuable resource for quality estimates during model selection.

To evaluate the per-interface predicted scores on CASP16 targets, we computed the average Spearman correlation, Pearson correlation, and Ranking Loss values (see Tables 4 and 5, available as supplementary data at *Bioinformatics* online), with each target-interface pair considered as an independent entry, and each entry is weighted by the number of interfaces in the respective target, for equal target representation. The ground truth quality is the per-interface ICS and IPS score. We ensured a fair comparison by setting Spearman and Pearson correlations to zero if they were negative. To reduce bias from incomplete data, the evaluation was only on target-interface pairs where all tools were able to predict at least 60% of the models. Individual metrics for each target-interface pair can be found in Figs 8 and 9, available as supplementary data at *Bioinformatics* online. CARP-predicted per-interface scores display clear advantages compared to other state-of-the-art tools. Our PerIF predicted scores ranked first for both IPS and ICS in terms of Ranking Loss. Our PerIF-G predicted scores ranked either first or second for all metrics, except for the IPS Spearman correlation. In particular, the superior performance of our PerIF-G in terms of correlation coefficients, which is an average of our predicted per-interface and global interface scores, suggests that global interface predictions can enhance per-interface quality estimates in real-world modeling scenarios.

Since human expert structural predictors predominantly performed the best (Kretsch et al. 2026) in CASP16 for the structure prediction of nucleic acid containing complexes, we investigated how CARP-selected models were generated. In Fig. 7, available as supplementary data at *Bioinformatics* online, we observed that human-guided deep learning methods were selected most frequently by CARP, and most deep learning methods used AF3 as the core of their pipeline. In this light, to assess CARP’s performance in re-scoring AF3 models for protein–RNA structure prediction, we created a custom dataset of AF3 models for the blind-test complexes. We generated a total of 100 models per complex (56 complexes in total) using 20 different seeds, each with 5 diffusion samples, to compare CARP’s scoring accuracy with that of other tools.

We used four metrics to assess the performance of the top-k ranked models, all of which are defined in terms of quantile:


(3) $$Q(x)=100\left(\frac{\text{count}(\text{iRMSD}\geq x)}{N_{\text{models}}}\right)$$

The quantile of a model, Q(x), refers to the percentage of models with an iRMSD value greater than or equal to the current model’s iRMSD (*x*), the details can be found in Supplementary 1.6.4, available as supplementary data at *Bioinformatics* online. True top models for a given complex are defined as those at or above the 99th quantile, Q(x)≥99. Success rate is the percentage of complexes where at least one top-k ranked model is a true top model, and recall is the percentage of the true top models selected within the top-k models, among all true top models. The average and best quantiles are the average and maximum quantiles, respectively, for the top-k selected models. For example, when k=5, the average quantile and best quantile are the mean and maximum quantiles for the top-5 selected models.

The average quantile (k=5) for all predictors, including the AlphaFold3 quality estimates, are shown in Fig. 12, available as supplementary data at *Bioinformatics* online. The average quantile, maximum quantile, success, and recall metrics when comparing against all other tools are shown in Figs 13–17, available as supplementary data at *Bioinformatics* online. From these results, it is evident that CARP shows improvement compared to all the other state-of-the-art tools when re-scoring AF3 models. We also evaluated CARP, based upon the same four metrics, with all 56 complexes, against AF3’s own estimates of ipTM and pTM scores, with the results shown in Fig. 11, available as supplementary data at *Bioinformatics* online. Our CARP-Merged Score achieved better performances than AF3 for all metrics with k=5. For all other values of *k* and metrics, our CARP-RP score achieved better performances than AF3. The detailed calculations for ipTM and pTM are described in Supplementary 2.3.1, available as supplementary data at *Bioinformatics* online.

We further evaluated the tools based on the docking blind-test dataset. Our method is comparable to others for ordering a set of docking decoys based on the interface quality (iRMSD). Since protein–RNA docking generates interface conformations using the native chains, we expected and observed that our overall fold predictor’s (CARP-Fold) performance is worse than that of our interface-focused predictors (CARP-RP, Merged, and Iface). For the top-5 selected decoys, our CARP-Merged score is better than all other tools for the average and best quantiles, except for FTDMP (Olechnovič et al. 2025). In terms of recall, our CARP-RP score is better than DRPScore (Zeng et al. 2023), 3dRPC (Li et al. 2017), DARS-RNP (Tuszynska and Bujnicki 2011), and QUASI-RNP (Tuszynska and Bujnicki 2011). However, as noted by Li et al. (2017), ITScore-PR (Huang and Zou 2014) excels at identifying top-ranked models when a near-native decoy is present, as evidenced by ITScore’s higher recall. In terms of success rate, our CARP-Merged score is better than both DRPScore (Zeng et al. 2023) and ITScore-PR (Huang and Zou 2014), whereas the remaining tools perform better than CARP quality estimates for docking success rates. These results, presented in Supplementary 2.4, available as supplementary data at *Bioinformatics* online, indicate that CARP can serve as a reliable complement to statistical potentials for docking.

To examine the interpretability of CARP, we performed feature sensitivity analysis, more details see Supplementary 2.5, available as supplementary data at *Bioinformatics* online. The boxen plots of the sensitivity scores for each feature are shown in Figs 35 and 36, available as supplementary data at *Bioinformatics* online, for CASP16 targets and the docking blind-test decoy complexes, respectively. It is clear from these plots that the different CARP scores respond differently to each feature, where RNA-related features correspond with relatively higher sensitivities for interface-focused scores (CARP-RP and CARP-Iface). Since CARP was trained using PyRosetta perturbed structures, we examined CARP’s outputs for perturbed blind-test docking decoys (complexes). Our findings demonstrate that CARP behaves as expected; details are provided in Supplementary 2.6, available as supplementary data at *Bioinformatics* online.

## 4 Conclusion

We developed CARP, which achieved significant improvement over existing tools for predicting the qualities of protein–RNA models. Our CARP-predicted scores are particularly useful for real-world model selection, as indicated by the CASP16 and AlphaFold3 performance analyses.

## Supplementary Material

btag202_Supplementary_Data

## Data Availability

The source code for the CARP software, along with the relevant datasets generated and analyzed during this study, is openly available on GitHub at github.com/zwang-bioinformatics/CARP/. Protein-RNA (hybrid) structure models from the CASP16 experiment are publicly accessible via the Protein Structure Prediction Center (predictioncenter.org). Native protein-RNA complex structures were obtained from the Protein Data Bank (www.rcsb.org).

## References

[btag202-B1] Abramson J , Adler J, Dunger J et al Accurate structure prediction of biomolecular interactions with AlphaFold 3. Nature 2024;630:493–500. 38718835 10.1038/s41586-024-07487-w PMC11168924

[btag202-B2] Biasini M , Schmidt T, Bienert S et al Openstructure: an integrated software framework for computational structural biology. Biol Crystallogr 2013;69:701–9. 10.1107/S0907444913007051 PMC3640466 23633579

[btag202-B3] Cai T , Luo S, Xu K et al Graphnorm: A principled approach to accelerating graph neural network training. In: *International Conference on Machine Learning*. Vienna, Austria: PMLR, 2021, 1204–15.

[btag202-B4] Chaudhury S , Lyskov S, Gray JJ. Pyrosetta: a script-based interface for implementing molecular modeling algorithms using Rosetta. Bioinformatics 2010;26:689–91. 20061306 10.1093/bioinformatics/btq007 PMC2828115

[btag202-B5] Chung C-S , Tseng C-K, Lai Y-H et al Dynamic protein–RNA interactions in mediating splicing catalysis. Nucleic Acids Res 2019;47:899–910. 30395327 10.1093/nar/gky1089 PMC6344849

[btag202-B6] Duarte CM , Pyle AM. Stepping through an RNA structure: a novel approach to conformational analysis. J Mol Biol 1998;284:1465–78. 9878364 10.1006/jmbi.1998.2233

[btag202-B7] Gao H , Ji S. Graph u-nets. IEEE Trans Pattern Anal Mach Intell 2019;44:4948–60. https://api.semanticscholar.org/CorpusID : 153311899 10.1109/TPAMI.2021.3081010 33999813

[btag202-B8] Glisovic T , Bachorik JL, Yong J et al RNA-binding proteins and post-transcriptional gene regulation. FEBS Lett 2008;582:1977–86. 18342629 10.1016/j.febslet.2008.03.004 PMC2858862

[btag202-B9] Høie MH , Kiehl EN, Petersen B et al NetSurfP-3.0: accurate and fast prediction of protein structural features by protein language models and deep learning. Nucleic Acids Res 2022;50:W510–5. 35648435 10.1093/nar/gkac439 PMC9252760

[btag202-B10] Hu W , Liu B, Gomes J et al Strategies for pre-training graph neural networks. In: *International Conference on Learning Representations*. Addis Ababa, Ethiopia. 2020. https://openreview.net/forum? id=HJlWWJSFDH

[btag202-B11] Huang S-Y , Zou X. A knowledge-based scoring function for protein–RNA interactions derived from a statistical mechanics-based iterative method. Nucleic Acids Res 2014;42:e55. 24476917 10.1093/nar/gku077 PMC3985650

[btag202-B12] Jumper J , Evans R, Pritzel A et al Highly accurate protein structure prediction with AlphaFold. Nature 2021;596:583–9. 34265844 10.1038/s41586-021-03819-2 PMC8371605

[btag202-B13] Kabsch W , Sander C. Dictionary of protein secondary structure: pattern recognition of hydrogen‐bonded and geometrical features. Biopolymers 1983;22:2577–637. 10.1002/bip.360221211 6667333

[btag202-B14] Ke A , Doudna JA. Crystallization of RNA and RNA–protein complexes. Methods 2004;34:408–14. 15325657 10.1016/j.ymeth.2004.03.027

[btag202-B15] Khalil AM , Rinn JL. RNA–protein interactions in human health and disease. Seminars Cell Dev Biol 2011;22:359–65. 10.1016/j.semcdb.2011.02.016 PMC3184770 21333748

[btag202-B16] Kretsch RC , Hummer AM, He S et al Assessment of nucleic acid structure prediction in CASP16. Proteins Struct Funct Bioinf 2026;94:192–217. 10.1002/prot.70072 PMC13185081 41165252

[btag202-B17] Leontis NB , Zirbel CL. Nonredundant 3D structure datasets for RNA knowledge extraction and benchmarking. In: Neocles Leontis, Eric Westhof (eds.), RNA 3D Structure Analysis and Prediction. Berlin, Heidelberg: Springer, 2012; 281–98.

[btag202-B18] Li H , Huang Y, Xiao Y. A pair-conformation-dependent scoring function for evaluating 3D RNA–protein complex structures. PLoS One 2017;12:e0174662. 28358834 10.1371/journal.pone.0174662 PMC5373608

[btag202-B19] Li Y , Zemel R, Brockschmidt M et al Gated graph sequence neural networks. In: *Proceedings of ICLR’16*. San Juan, Puerto Rico, 2016. https://www.microsoft.com/en-us/research/publication/gated-graph-sequence-neural-networks/

[btag202-B20] Mirdita M , Schütze K, Moriwaki Y et al ColabFold: making protein folding accessible to all. Nat Methods 2022;19:679–82. 10.1038/s41592-022-01488-1 35637307 PMC9184281

[btag202-B21] Ning S , Zeng C, Zeng C et al The TAR binding dynamics and its implication in Tat degradation mechanism. Biophys J 2021;120:5158–68. 34762866 10.1016/j.bpj.2021.11.006 PMC8715215

[btag202-B22] Olechnovič K , Banciul R, Dapkūnas J et al FTDMP: a framework for protein–protein, protein–DNA, and protein–RNA docking and scoring. Proteins Struct Funct Bioinf 2025. 10.1002/prot.26792 39748638

[btag202-B23] Olechnovič K , Venclovas Č. VoroIF-GNN: Voronoi tessellation-derived protein–protein interface assessment using a graph neural network. Proteins 2023;91:1879–88. 37482904 10.1002/prot.26554

[btag202-B24] Ontiveros-Palacios N , Cooke E, Nawrocki EP et al Rfam 15: RNA families database in 2025. Nucleic Acids Res 2025;53:D258–67. 39526405 10.1093/nar/gkae1023 PMC11701678

[btag202-B25] Qiu L , Zou X. Scoring functions for protein–RNA complex structure prediction: advances, applications, and future directions. Commun Inf Syst 2020;20:1–22. 33867869 10.4310/cis.2020.v20.n1.a1 PMC8049283

[btag202-B26] Ramanathan M , Porter DF, Khavari PA. Methods to study RNA–protein interactions. Nat Methods 2019;16:225–34. 30804549 10.1038/s41592-019-0330-1 PMC6692137

[btag202-B27] Rampášek L , Galkin M, Dwivedi VP et al Recipe for a general, powerful, scalable graph transformer. Adv Neural Inf Process Syst 2022;35:14501–15.

[btag202-B28] Réau M , Renaud N, Xue LC et al DeepRank-GNN: a graph neural network framework to learn patterns in protein–protein interfaces. Bioinformatics 2023;39:btac759. 36420989 10.1093/bioinformatics/btac759 PMC9805592

[btag202-B29] Sato K , Kato Y, Hamada M et al IPknot: fast and accurate prediction of RNA secondary structures with pseudoknots using integer programming. Bioinformatics 2011;27:i85–93. 21685106 10.1093/bioinformatics/btr215 PMC3117384

[btag202-B30] Schrödinger LLC. *The PyMOL Molecular Graphics System, Version 2.5.4*. 2015.

[btag202-B31] Scott LG , Hennig M. RNA structure determination by NMR. Bioinf Data Sequence Anal Evol 2008;452:29–61. 10.1007/978-1-60327-159-2_2 18563368

[btag202-B32] Shi Y , Huang Z, Feng S et al Masked label prediction: Unified message passing model for semi-supervised classification. In: *Proceedings of the Thirtieth International Joint Conference on Artificial Intelligence (IJCAI 2021), Main Track*. Montreal, Canada. 2021;1548–54. 10.24963/ijcai.2021/214

[btag202-B33] Steinegger M , Söding J. Mmseqs2 enables sensitive protein sequence searching for the analysis of massive data sets. Nat Biotechnol 2017;35:1026–8. 29035372 10.1038/nbt.3988

[btag202-B34] Studer G , Tauriello G, Schwede T. Assessment of the assessment—all about complexes. Proteins Struct Funct Bioinf 2023;91:1850–60. 10.1002/prot.26612 37858934

[btag202-B35] Thiel BC , Beckmann IK, Kerpedjiev P et al 3D based on 2D: Calculating helix angles and stacking patterns using forgi 2.0, an RNA Python library centered on secondary structure elements. F1000Research, 2019;8. 10.12688/f1000research.18458.1 PMC6480952 31069053

[btag202-B36] Tuszynska I , Bujnicki JM. DARS-RNP and QUASI-RNP: new statistical potentials for protein–RNA docking. BMC Bioinformatics 2011;12:1–16. 21851628 10.1186/1471-2105-12-348 PMC3179970

[btag202-B37] Vaswani A , Shazeer N, Parmar N et al Attention is all you need. Adv Neural Inf Process Syst 2017;30;5998–6008.

[btag202-B38] Watkins AM , Rangan R, Das R. FARFAR2: improved de novo Rosetta prediction of complex global RNA folds. Structure 2020;28:963–76.e6. 32531203 10.1016/j.str.2020.05.011 PMC7415647

[btag202-B39] Yang H , Jossinet F, Leontis N et al Tools for the automatic identification and classification of RNA base pairs. Nucleic Acids Res 2003;31:3450–60. 12824344 10.1093/nar/gkg529 PMC168936

[btag202-B40] Zeng C , Jian Y, Vosoughi S et al Evaluating native-like structures of RNA–protein complexes through the deep learning method. Nat Commun 2023;14:1060. 36828844 10.1038/s41467-023-36720-9 PMC9958188

[btag202-B41] Zeng C , Zhuo C, Gao J et al Advances and challenges in scoring functions for RNA–protein complex structure prediction. Biomolecules 2024;14:1245. 39456178 10.3390/biom14101245 PMC11506084

[btag202-B42] Zhang C , Shine M, Pyle AM et al US-align: universal structure alignments of proteins, nucleic acids, and macromolecular complexes. Nat Methods 2022;19:1109–15. 36038728 10.1038/s41592-022-01585-1

[btag202-B43] Zhang H , Zhang L, Mathews DH et al LinearPartition: linear-time approximation of RNA folding partition function and base-pairing probabilities. Bioinformatics 2020;36:i258–67. 32657379 10.1093/bioinformatics/btaa460 PMC7355276

