# Supplementary material for: Inferring the qualities of protein–RNA models with graph transformers
Source: Bioinformatics. 2026 Apr 28;42(5):btag202. doi: 10.1093/bioinformatics/btag202 (PMC13189855; doi:10.1093/bioinformatics/btag202)
Supplement: btag202_Supplementary_Data [file btag202_supplementary_data.zip › supplementary.pdf]

Supplementary document

Inferring the qualities of protein-RNA models with graph transformers

Andrew Jordan Siciliano[1], Yifan Bao[1], Bishal Shrestha[1], and Zheng Wang[1,*]

[1]Department of Computer Science, University of Miami, 1365 Memorial Drive, Coral Gables, 33124, FL, USA
[*]Correspondence: zheng.wang@miami.edu

Contents

S.1 Supplementary methods **2**
S.1.1 Implementation details . . . 2
S.1.2 Decoy generation . . . 2
S.1.3 Features and algorithm . . . 2
S.1.4 Loss function . . . 3
S.1.5 Learning curves . . . 3
S.1.6 Structure comparison scores . . . 7
S.1.6.1 TM-score . . . 7
S.1.6.2 lDDT and variants . . . 7
S.1.6.3 Oligo-GDTTS and Oligo-GDTHA . . . 7
S.1.6.4 RMSD, iRMSD, and iRMSD variants . . . 8
S.1.6.5 IPS and ICS . . . 8
S.1.7 Predicted CARP quality scores formulae . . . 8
S.1.8 Predicted quality (and energy) scores descriptions . . . 8

S.2 Supplementary results **9**
S.2.1 CASP16 results . . . 9
S.2.2 Blind-test target filtering . . . 15
S.2.3 AlphaFold3 results . . . 15
S.2.3.1 Comparison with ipTM and pTM . . . 15
S.2.3.2 Comparison with other tools for Average Quantile, Best Quantile, Success, and Recall . . 16
S.2.3.3 Per-complex kernel density estimation and rankings . . . 19
S.2.4 Docking results . . . 25
S.2.4.1 Average Quantile, Best Quantile, Recall, and Success . . . 25
S.2.4.2 Per-complex kernel density estimation and rankings . . . 28
S.2.5 Feature analysis . . . 34
S.2.6 Docking perturbation analysis . . . 35

References **37**

S.1 Supplementary methods

S.1.1 Implementation details

CARP is implemented in Python using PyTorch [1] and PyTorch Geometric [2]. We trained our models on a NVIDIA A100 GPU. Deep learning models were optimized using the AdamW optimizer [3], and gradients were adaptively clipped using AutoClip [4].

S.1.2 Decoy generation

For each protein-RNA complex (150), 24 perturbed native protein-RNA complexes were included in the training set. For each of protein-RNA complex, we also generated 10,000 decoys (when possible). Due to technical constraints, our structure-generation pipeline failed to produce complete decoy sets for all complexes. These failures primarily occurred when 3dRPC [5] terminated due to large grid sizes (memory constraints) and/or large polymer sizes. Rare issues with PyRosetta [6] (on the 3dRPC decoys) and OpenStructure [7] also contributed to missing data. Issues only occurred in 16 of the 150 protein-RNA complexes. For the 134 complexes without generation issues, each included 500 3dRPC decoys (closest to the native structure in terms of RMSD), along with 2400 perturbed 3dRPC decoys, corresponding to 24 perturbations for each of the 100 closest 3dRPC decoys to the native structure in terms of RMSD.

S.1.3 Features and algorithm

Algorithm S.1 Deep learning pipeline.

Input: $(X \in \mathbb{R}^{N\times 66},\ E \in \mathbb{R}^{L\times 12},\ A \in \{0,1\}^{N\times N}) \sim G_0$
$G_1 \leftarrow \text{TopK} \leftarrow \text{ReLU} \leftarrow \text{GraphNorm} \leftarrow \textbf{GPSConv}(G_0)$
$G_2 \leftarrow \text{TopK} \leftarrow \text{ReLU} \leftarrow \text{GraphNorm} \leftarrow \textbf{TransformerConv}(G_1)$
$\tilde{G}_2 \leftarrow \text{rpFilt}(G_2)$ ▷ remove non protein-RNA interactions
$(X_3, E_3, A_3) \sim G_3 \leftarrow \text{TopK} \leftarrow \text{ReLU} \leftarrow \text{GraphNorm} \leftarrow \textbf{TransformerConv}(\tilde{G}_2)$
$G_3 \leftarrow (\text{Cat}([X_3, \text{Polymer Type}, \text{Is Interface (rp) Node}]), E_3, A_3)$

Output Predictions:
$(\hat{F}_0, \hat{F}_1, \hat{F}_2) \leftarrow (\text{bb-LDDT, Oligo-GDTTS, Oligo-GDTHA})_{\text{pred}} \leftarrow \textbf{GAP}(G_3)$ ▷ Global Fold Pooling
$(\hat{I}_0, \hat{I}_1, \hat{I}_2) \leftarrow (\text{iLDDT, IPS, ICS})_{\text{pred}} \leftarrow \textbf{GAP}(G_3)$ ▷ Global Interface Pooling
$(\hat{P}_0[i], \hat{P}_1[i]) \leftarrow (\text{IPS, ICS})_{\text{pred}[i]} \leftarrow \textbf{GAP}(G_3)$ ▷ Per-Interface Pooling

Table S.1: Node and edge Features

Category	Tool/s	Description	Source	Channels
Node		Residue Type	Sequence	26
Node		Polymer Type	Sequence	1
Node	IPknot [8]	*Predicted:*<br>Interacting Nucleotide	Sequence	1
Node	NetSurfP [9]	*Predicted:*<br>Protein RSA & Torsion Angles (ψ, ϕ)<br>Protein Secondary Structure<br>Protein Disorder	Sequence	9
Node		Is Interface (rp) Node	Model	1
Node		Center of Mass Distances	Model	5
Node		Center of Mass Angles	Model	4
Node	AMIGOS [10]	*Annotated:*<br>RNA Torsion Angles (η, θ)	Model	4
Node	RNAView [11]<br>Forgi [12]	*Annotated:*<br>Interacting Nucleotide	Model	2
Node	DSSP [13]	*Annotated:*<br>Protein Secondary Structure<br>Protein RSA & Torsion Angles (ψ, ϕ)	Model	13
Edge		Is Inter-Chain	Model	1
Edge		Is Protein-RNA Interaction	Model	1
Edge		Nearest Atom-Atom Distance	Model	1
Edge		Backbone Distance	Model	1
Edge		Backbone Angle	Model	2
Edge	RNAView [11]	*Annotated:*<br>RNA Base-Pair Interaction	Model	1
Edge	AlphaFold2 [14, 15]	*Predicted:*<br>Protein Intra Residue-Residue Distance<br>Protein Intra Residue-Residue Angle	Sequence	3
Edge	LinearPartition [16]	*Predicted:*<br>RNA Base-Pair Interaction Probability	Sequence	1
Edge	IPknot [8]	*Predicted:*<br>RNA Base-Pair Interaction	Sequence	1

S.1.4 Loss function

$$\mathcal{L}_{\text{global}} = \frac{1}{6} \sum_{k=1}^{3} |F_k - \hat{F}_k| + |I_k - \hat{I}_k|$$

$$\mathcal{L}_{\text{interface}} = \frac{1}{2\,(\#\text{Iface})} \sum_{i} \sum_{k \in \{0,1\}} |P_k[i] - \hat{P}_k[i]|$$

$$\mathcal{L}_{\text{combined}} = \begin{cases} \frac{\mathcal{L}_{\text{global}} + \mathcal{L}_{\text{interface}}}{2} & \text{if } \#\text{Iface} > 0 \\ \mathcal{L}_{\text{global}} & \text{else} \end{cases}$$

S.1.5 Learning curves

Figure S.1: Learning curve for Fold-1.

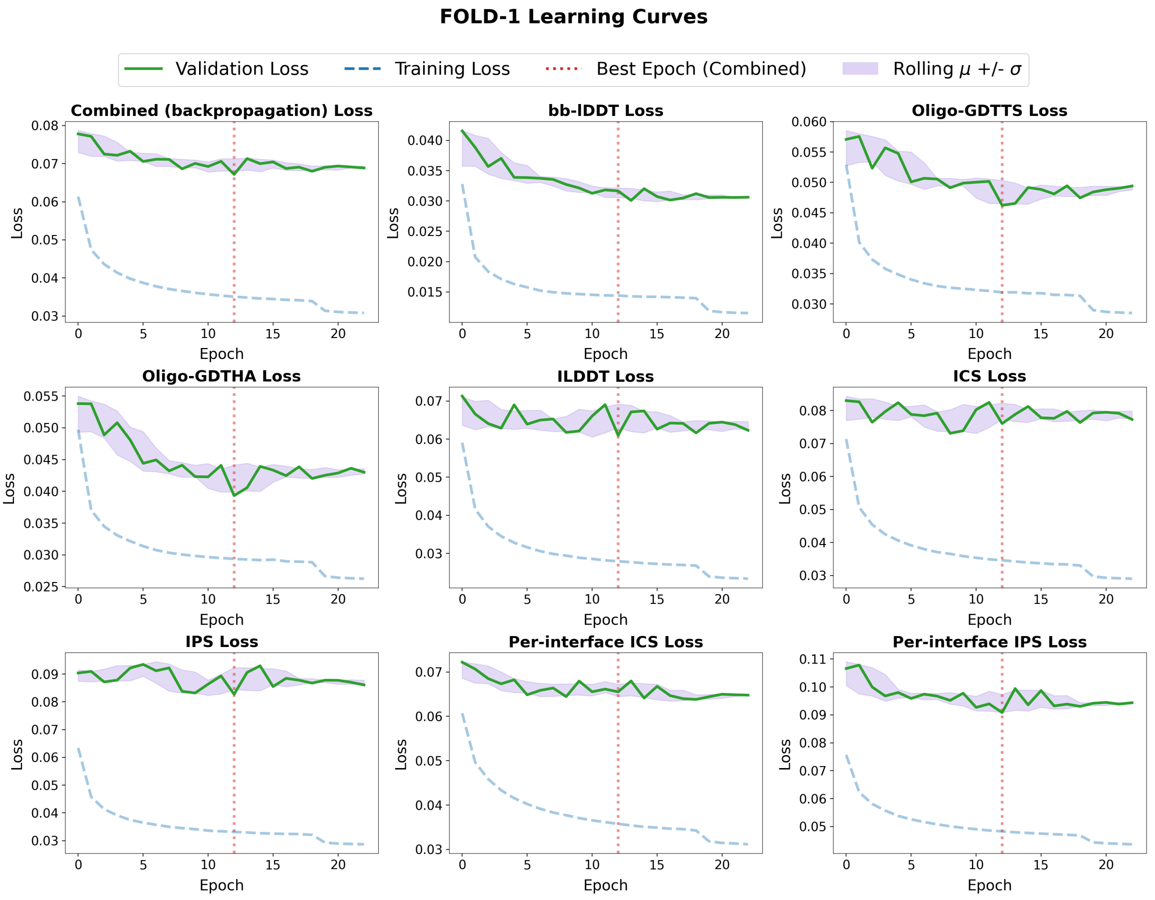

Figure S.2: Learning curve for Fold-2.

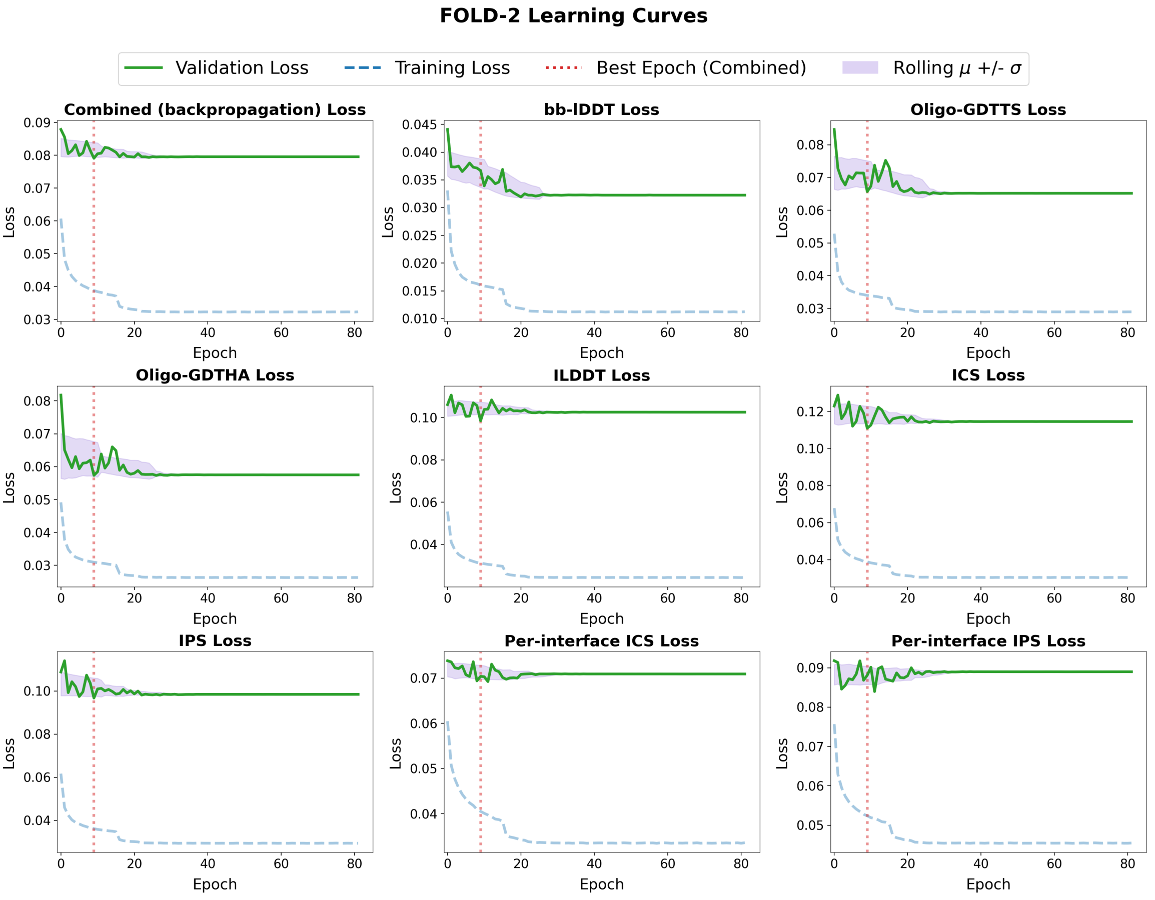

Figure S.3: Learning curve for Fold-3.

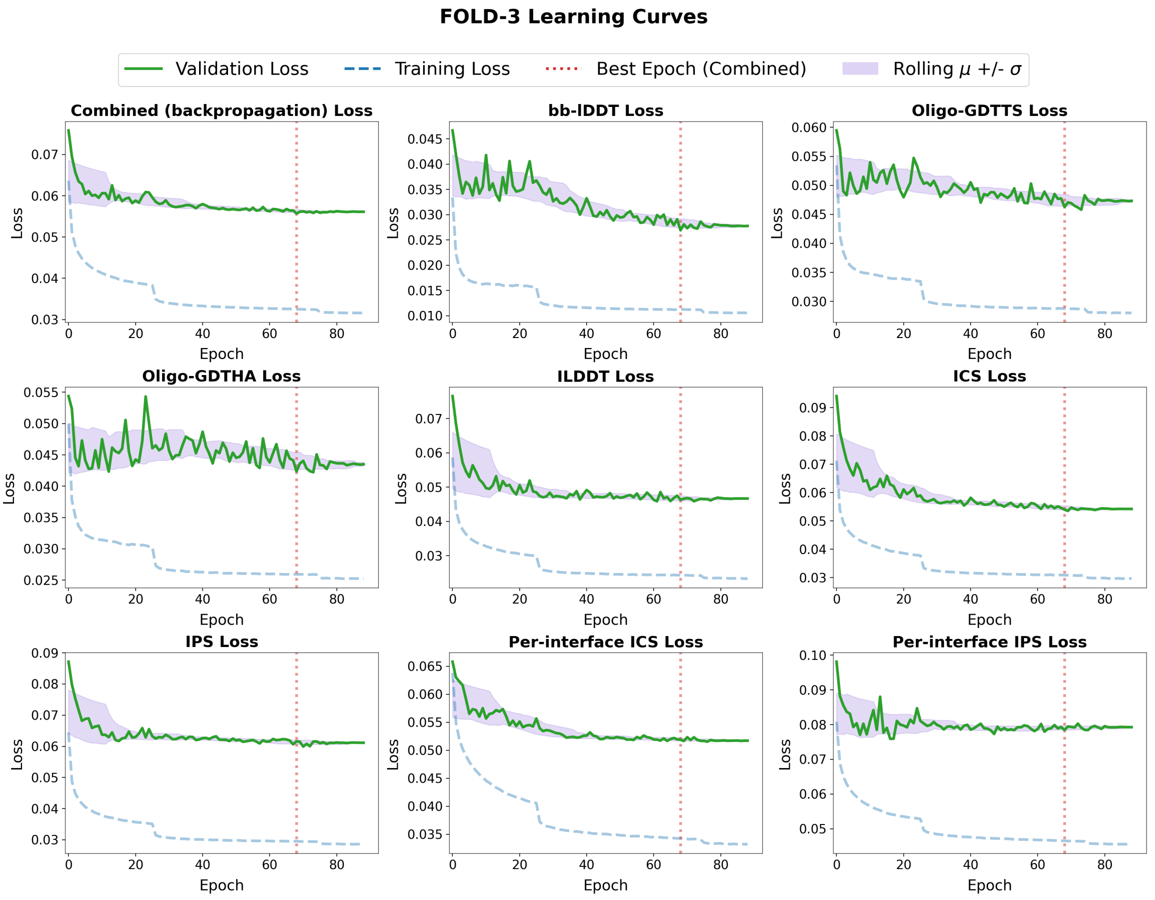

Figure S.4: Learning curve for Fold-4.

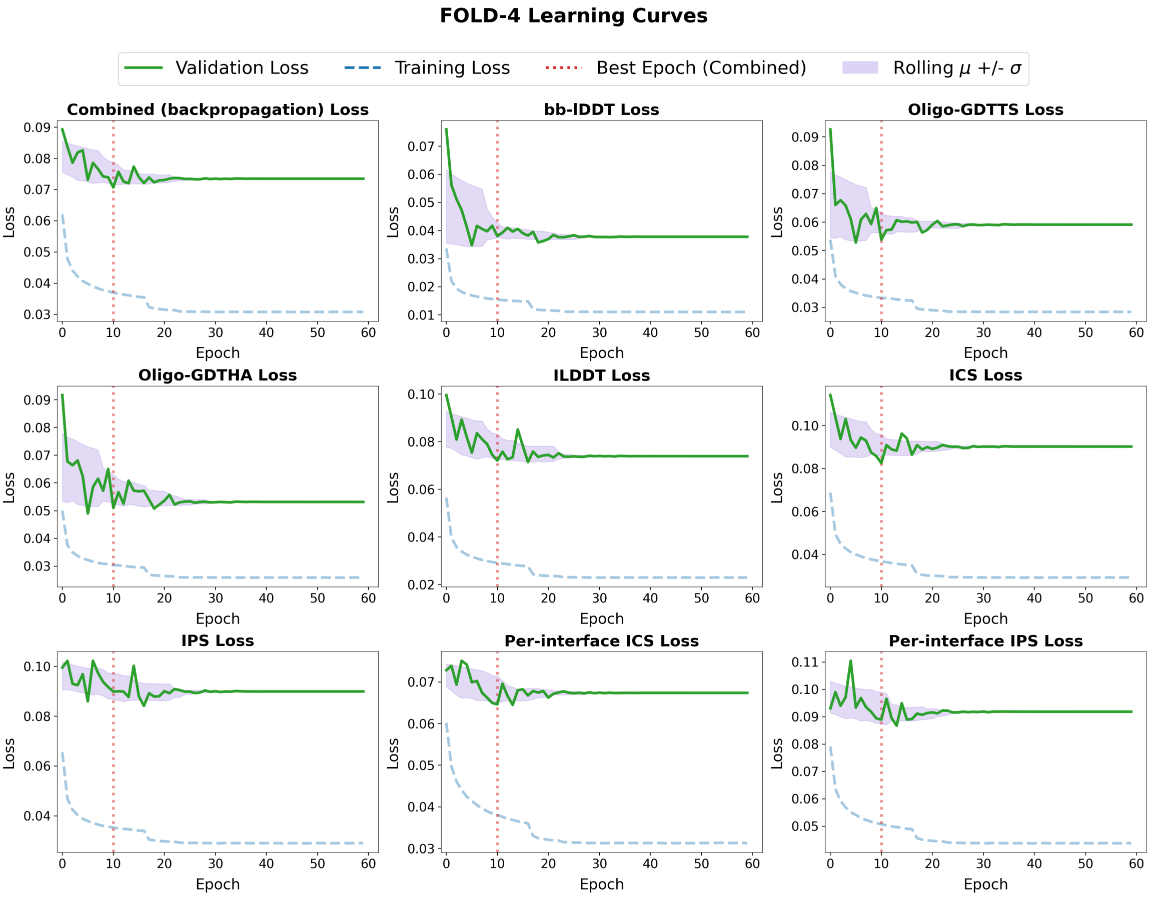

Figure S.5: Learning curve for Fold-5.

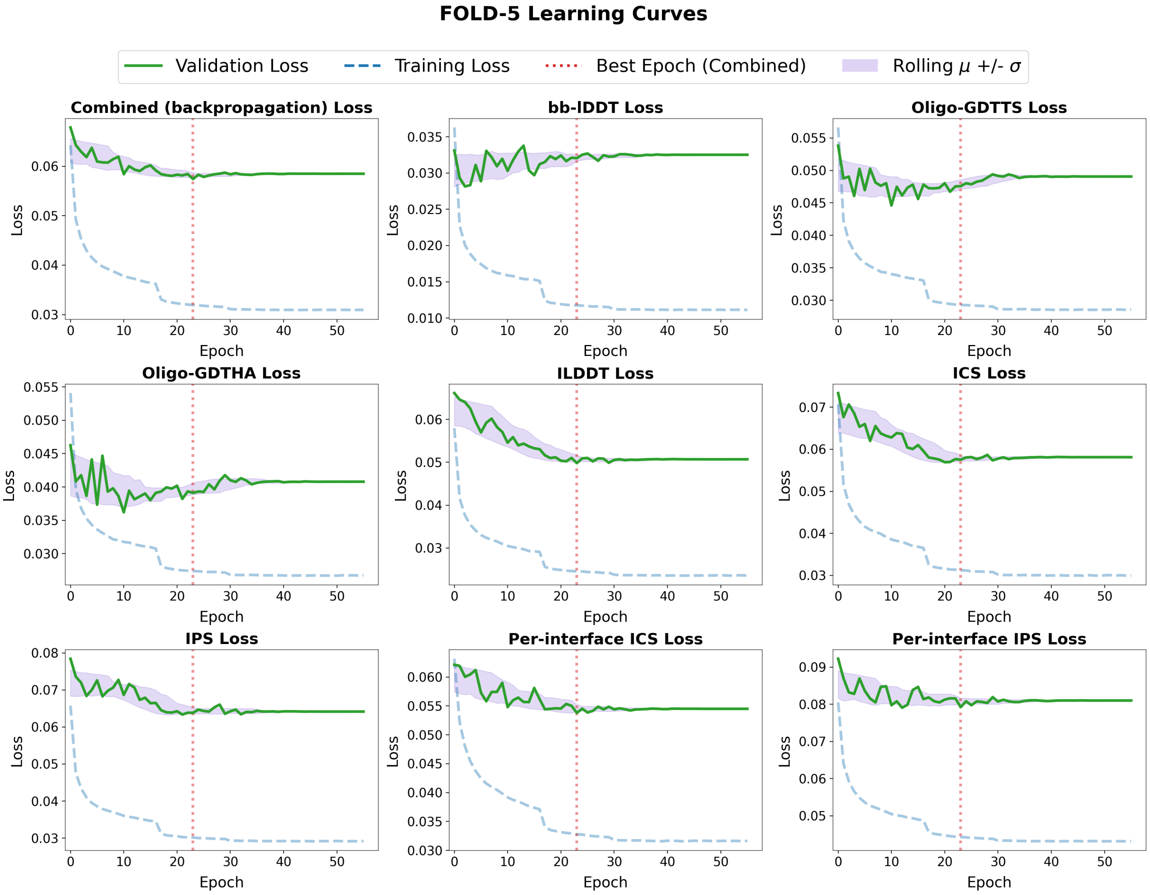

S.1.6 Structure comparison scores

Table S.2: Quality scores used for evaluating protein-RNA complex models.

Score	Focus	Tool	Ground truth for training	Released by CASP16
bb-LDDT	global-fold	OpenStructure [7]	Yes (F_0)	Yes (LDDT)
Oligo-GDTTS	global-fold	OpenStructure [7]	Yes (F_1)	Yes
Oligo-GDTHA	global-fold	OpenStructure [7]	Yes (F_2)	No
TM-score	global-fold	OpenStructure [7]	No	Yes
RMSD	global-fold	OpenStructure [7]	No	Yes
iLDDT	global-interface	OpenStructure [7]	Yes (I_0)	Yes
IPS	global-interface	OpenStructure [7]	Yes (I_1)	Yes
ICS	global-interface	OpenStructure [7]	Yes (I_2)	Yes
iRMSD	global-interface	Custom script [7]	No	No
IPS	per-interface	OpenStructure [7]	Yes ($P_0[i]$)	No
ICS	per-interface	OpenStructure [7]	Yes ($P_1[i]$)	Yes

S.1.6.1 TM-score

The formula for TM-score [17] is defined as:

$$\text{TM-score} = \max_{\text{alignments}} \left(\frac{1}{L_t} \sum_{i}^{L_c} \frac{1}{1 + \left(\frac{d_i}{d_0(L_t)}\right)^2} \right)$$

The length of the reference protein is L_t, and the length of the paired aligned residues between the model and reference is L_c. The distance scale, for a given length, as defined by [17], is:

$$d_0(L) = 1.24\,(L - 15)^{\frac{1}{3}} - 1.8$$

S.1.6.2 lDDT and variants

The formula for lDDT [18] is defined as:

$$\text{lDDT} = \frac{1}{4} \sum_{d \in \{0.5,1,2,4\}} \frac{N_c(d)}{N_t}$$

$N_c(d)$ is the number of distances between residues whose distance is within dÅ of the reference's pairwise distance (excluding distances larger than 15Å), and N_t is the total number of distances $\leq$ 15Å. Similarly, for ilDDT, the distances are restricted to the subset of interface interactions. More details can be found in the OpenStructure [7] documentation.

S.1.6.3 Oligo-GDTTS and Oligo-GDTHA

Oligo-GDTTS and Oligo-GDTHA [19, 20, 21] are defined in terms of the Euclidean distance between each residue in the model ($\mathcal{M}$) after superimposition with the reference ($\mathcal{R}$) structure. The number of aligned residues is denoted as L.

$$\text{GDT}(d) = \frac{1}{L} \sum_{i=1}^{L} \mathbb{1}(||\mathcal{M}[i] - \mathcal{R}[i]||_2 \leq d)$$

Then, we can define the full formula for Oligo-GDTT as:

$$\text{Oligo-GDTTS} = \frac{1}{4} \sum_{d \in \{1,2,4,8\}} \text{GDT}(d)$$

The more strict score, Oligo-GDTHA, is defined using more stringent distance thresholds:

$$\text{Oligo-GDTHA} = \frac{1}{4} \sum_{d \in \{0.5,1.0,2.0,4.0\}} \text{GDT}(d)$$

S.1.6.4 RMSD, iRMSD, and iRMSD variants

Let us define the superimposed (SVDSuperimpositon) [7] and aligned reference and model as $\mathcal{M} \in \mathbb{R}^{N\times 3}$ and $\mathcal{R} \in \mathbb{R}^{N\times 3}$, respectively. Coordinates for nucleotides are set as C3' (for iRMSD C4') position, and for amino acids, it is $C\alpha$. The general formula for the RMSD between the reference and the model, after superimposition, is defined as:

$$\text{RMSD} = \sqrt{\frac{1}{N}\sum_{i=1}^{N} ||\mathcal{R}[i] - \mathcal{M}[i]||_2^2}$$

To compute the interface RMSD, inspired by [22, 23, 24], we defined two variants of iRMSD. The first is computing the RMSD using the optimal rigid transformation (rotation and translation) for aligning only protein residues. This rigid transformation is applied to all atoms, and the iRMSD is computed over all interface residues and any nucleotide-amino acid pair ($C\alpha$ and $C4'$ for amino acids and nucleotides, respectively) that is within 10.0 Å of one another in the reference structure. The second method involved superimposing the interface residues directly onto the reference, i.e., superimposing only the protein-RNA interface residues. In both cases, the iRMSD is computed using only the interface residues; the difference lies in which subset of residues the superimposition is performed on.

S.1.6.5 IPS and ICS

IPS (interface patch similarity) [25], as per the OpenStructure [7] documentation, is the Jaccard similarity coefficient between the set of interface residues in the reference structure and the model structure.

$$\text{IPS} = \frac{|\text{if}(\mathcal{R}) \cap \text{if}(\mathcal{M})|}{|\text{if}(\mathcal{R}) \bigcup \text{if}(\mathcal{M})|}$$

ICS (interface contact similarity) [25], as per the OpenStructure [7] documentation, is the F1-score of the aligned interface contacts that are present in the model and the interface contacts that are present in the reference. A contact is defined between residues if any heavy atom between the two residues is within 5 Å of one another.

$$\text{ICS} = \frac{2|\text{Cont}(\mathcal{R}) \cap \text{Cont}(\mathcal{M})|}{|\text{Cont}(\mathcal{R})| + |\text{Cont}(\mathcal{M})|}$$

S.1.7 Predicted CARP quality scores formulae

$$\text{Fold} = \frac{\hat{F}_0 + \hat{F}_1 + \hat{F}_2}{3} \tag{S.1}$$

$$\text{Iface} = \frac{\hat{I}_0 + \hat{I}_1 + \hat{I}_2}{3} \tag{S.2}$$

$$\text{Merged} = \frac{\text{Iface} + \text{Fold}}{2} \tag{S.3}$$

$$\text{PerIF}(i) = \frac{\hat{P}_0[i] + \hat{P}_1[i]}{2} \tag{S.4}$$

$$\text{PerIF-G}(i) = \frac{\text{PerIF}(i) + \text{Iface}}{2} \tag{S.5}$$

$$\text{RP-Iface} = \left(\frac{1}{\#\text{Iface}}\right)\sum_i \left(\frac{\hat{P}_0[i] + \hat{P}_1[i]}{2}\right) \tag{S.6}$$

S.1.8 Predicted quality (and energy) scores descriptions

Table S.3: Quality score descriptions for our tool and the tools used for comparison.

Tool	Global Quality	Per-Interface (rp) Quality
CARP	*Iface*, *Fold*, *Merged*, and *RP*	*PerIF* and *PerIF-G*
DRPScore [24]	*Avg* ($\log_{10}$ P(Native))	$\log_{10}$ P(Native)
ITScorePR [22]	$-E$	$-E$ (each rp dimer)
3dRPC-Score [5]	$-E$	$-E$ (each rp dimer)
FTDMP [26]	−gen_voromqa_energy	−gen_voromqa_energy (each rp dimer)
QUASI-RNP [27]	$-E$	$-E$ (each rp dimer)
DARS-RNP [27]	$-E$	$-E$ (each rp dimer)

S.2 Supplementary results

S.2.1 CASP16 results

For CASP16 targets, we were able to access the reference structures for all but two targets, M1221 and M1224, for which ground truth scores were obtained solely from the CASP16 website. For all targets except these, we reran OpenStructure using our custom script and calculated the chain-mapping and respective per-interface IPS and ICS scores for evaluations.

Table S.4: Average per-interface ICS evaluation results on CASP16 targets.

Method	Spearman Correlation	Pearson Correlation	Ranking Loss
CARP-PerIF*	0.104	0.117	**0.264**
CARP-PerIF-G*	**0.236**	**0.315**	0.361
DRPScore	0.155	0.190	0.524
ITScore-PR	0.198	0.169	0.463
3dRPC	0.150	0.081	0.541
FTDMP	0.119	0.079	0.482
QUASI-RNP	0.126	0.106	0.538
DARS-RNP	0.157	0.158	0.507

Table S.5: Average per-interface IPS evaluation results on CASP16 targets.

Method	Spearman Correlation	Pearson Correlation	Ranking Loss
CARP-PerIF*	0.142	0.177	**0.297**
CARP-PerIF-G*	0.236	**0.378**	0.365
DRPScore	0.116	0.219	0.544
ITScore-PR	**0.296**	0.277	0.477
3dRPC	0.202	0.112	0.577
FTDMP	0.141	0.117	0.533
QUASI-RNP	0.209	0.177	0.576
DARS-RNP	0.246	0.242	0.555

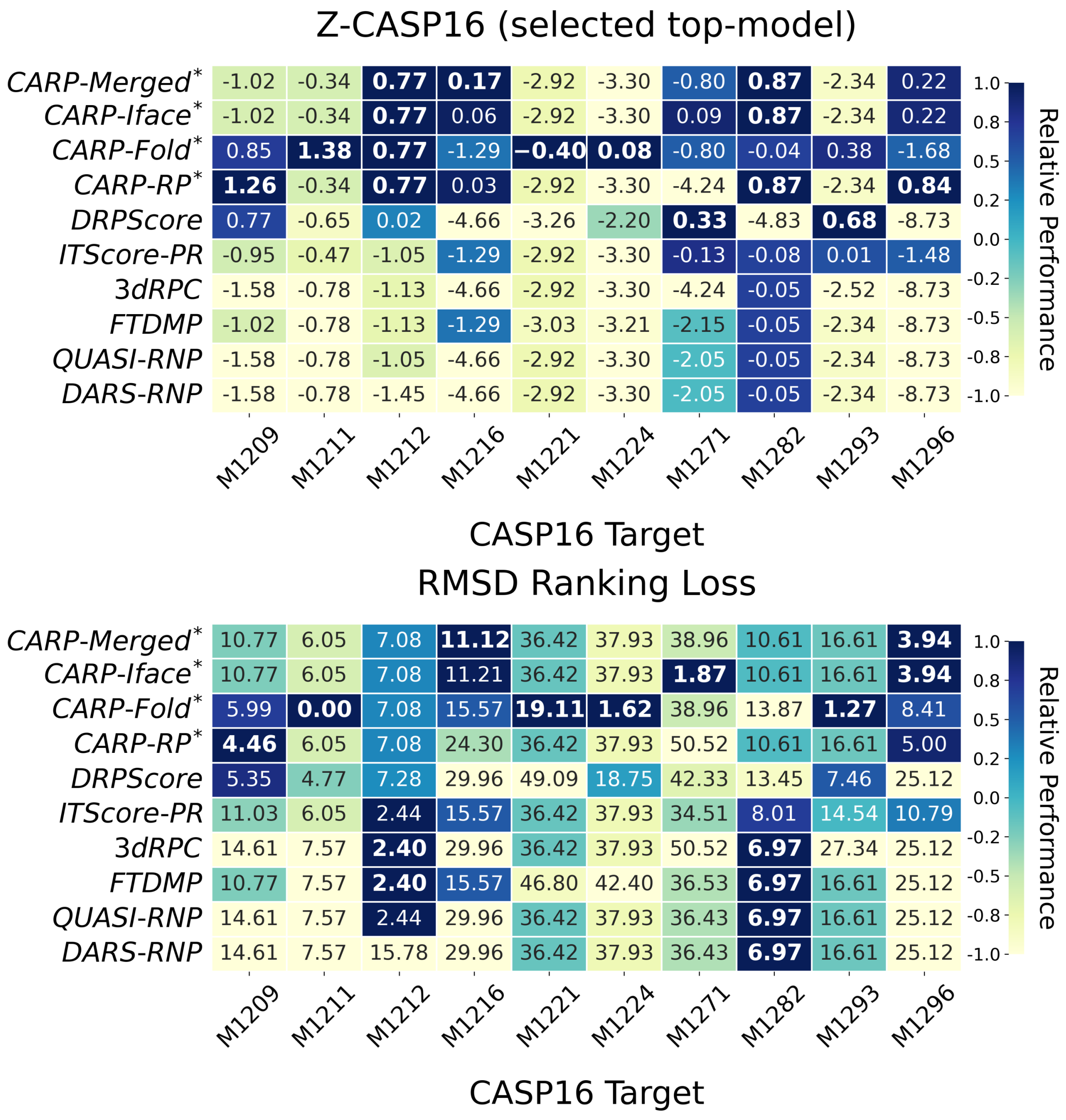

Figure S.6: Top-selected model's Z-CASP16 score (A) and RMSD Ranking Loss (B) for CASP16 targets. The color characterizes the relative performance of a method (min-max scaled), with darker colors indicating stronger performance.

Figure S.7: CASP16 Structure Prediction Groups Well-Ranked by CARP scores. The occurrences are counted over the top-3 selected models.

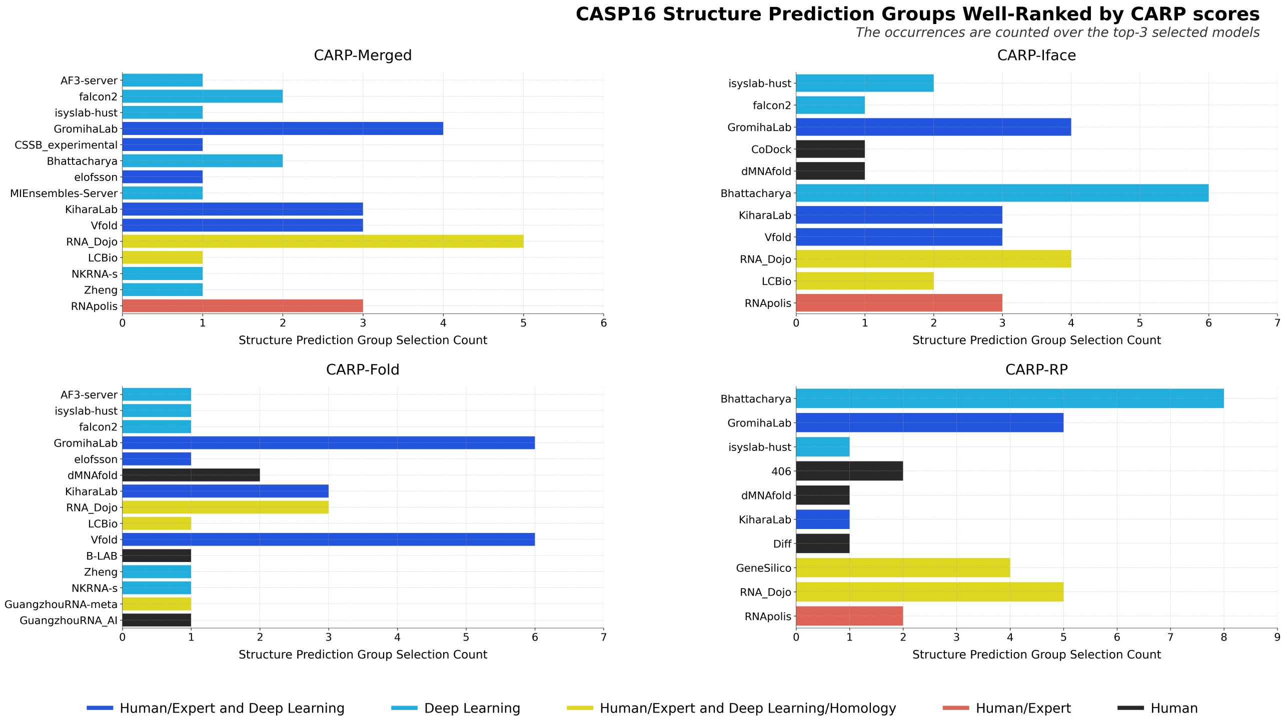

Figure S.8: Per-interface results for each target-interface pair, in terms of Pearson correlation (A), Spearman correlation (B), and Ranking Loss (C), with the ground truth quality of per-interface ICS score. The evaluation was exclusively on target-interface pairs, where all methods were able to predict for at least 60 percent of the models. (*) indicates one of our CARP predicted per-interface scores. The color characterizes the relative performance of a method (min-max scaled), with darker colors indicating stronger performance.

(A)

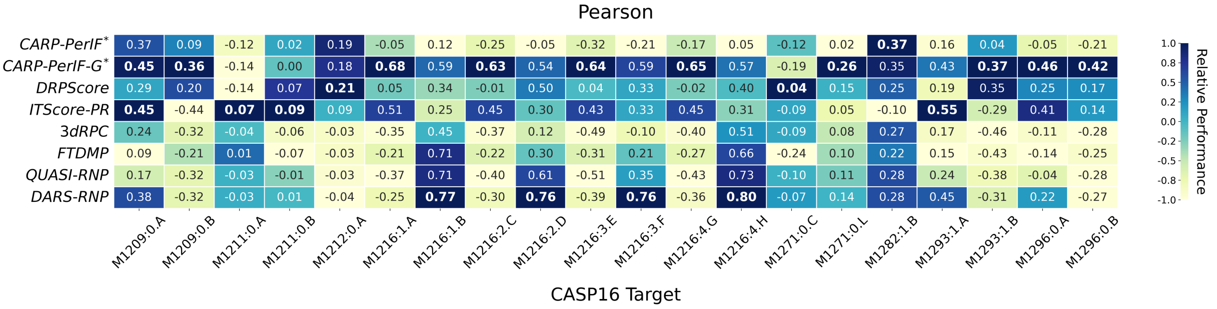

(B)

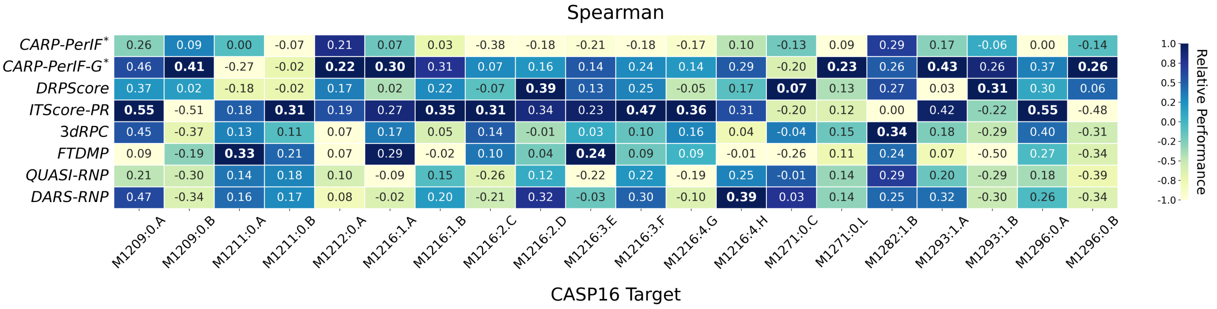

(C)

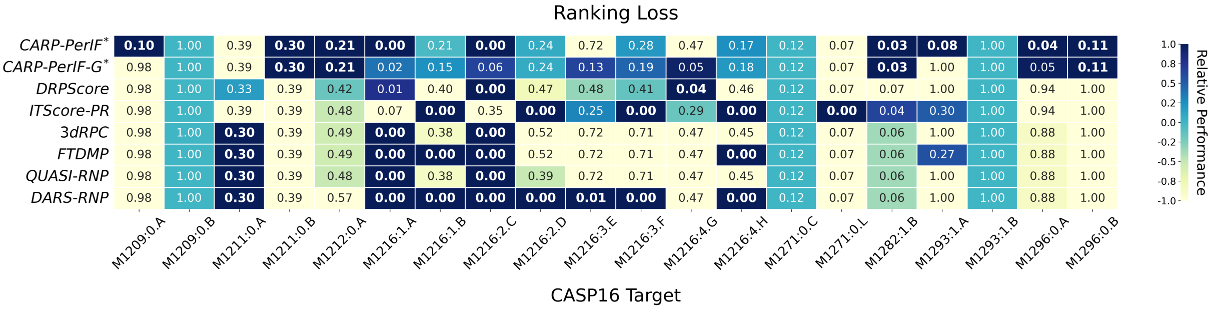

Figure S.9: Per-interface results for each target-interface pair, in terms of Pearson correlation (A), Spearman correlation (B), and Ranking Loss (C), with the ground truth quality of per-interface IPS score. The evaluation was exclusively on target-interface pairs, where all methods were able to predict for at least 60 percent of the models. (*) indicates one of our CARP predicted per-interface scores. The color characterizes the relative performance of a method (min-max scaled), with darker colors indicating stronger performance.

(A)

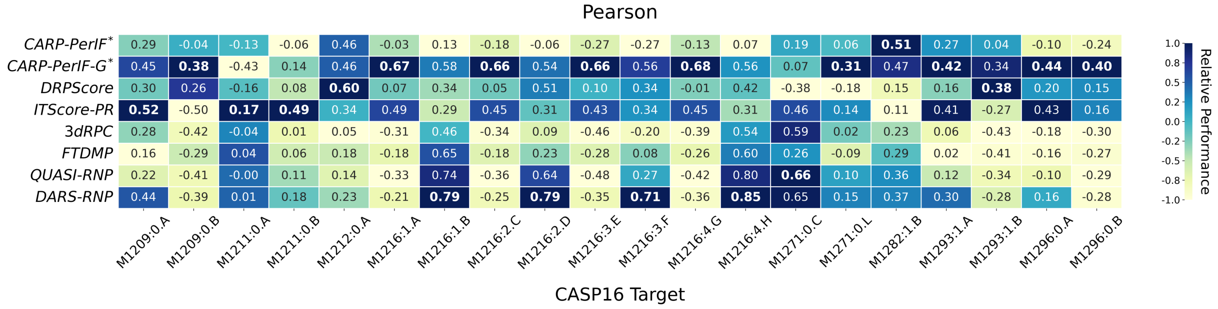

(B)

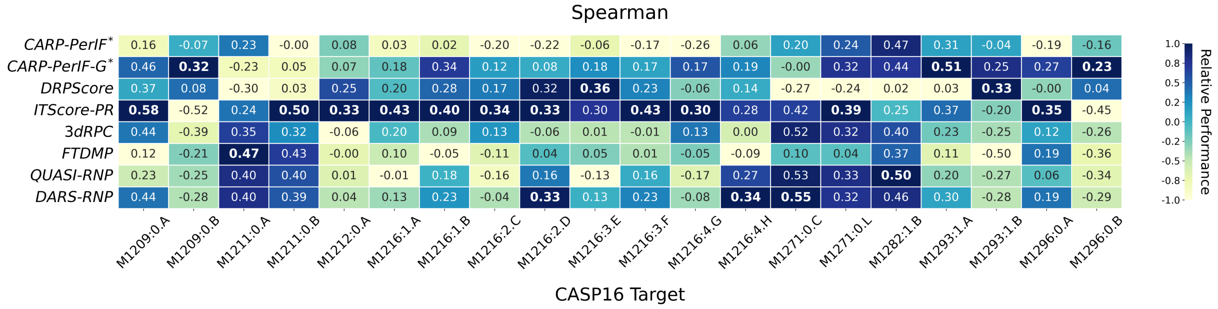

(C)

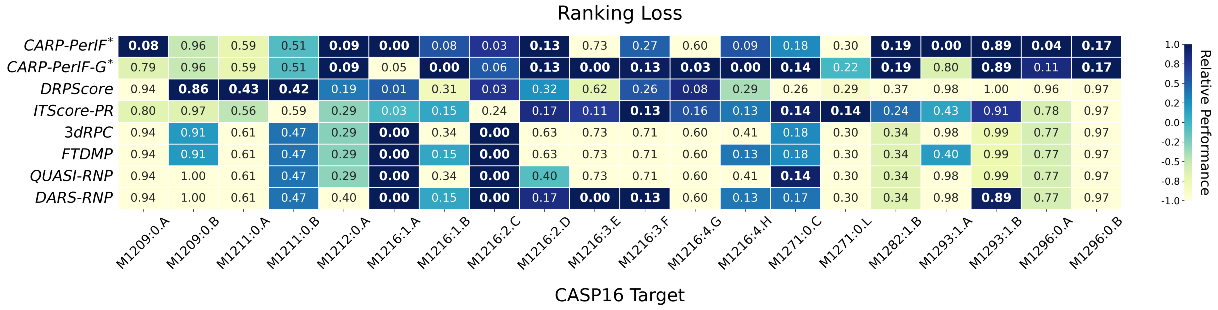

Figure S.10: Density (KDE) and predictor rankings per-target for Z-CASP16 scores.

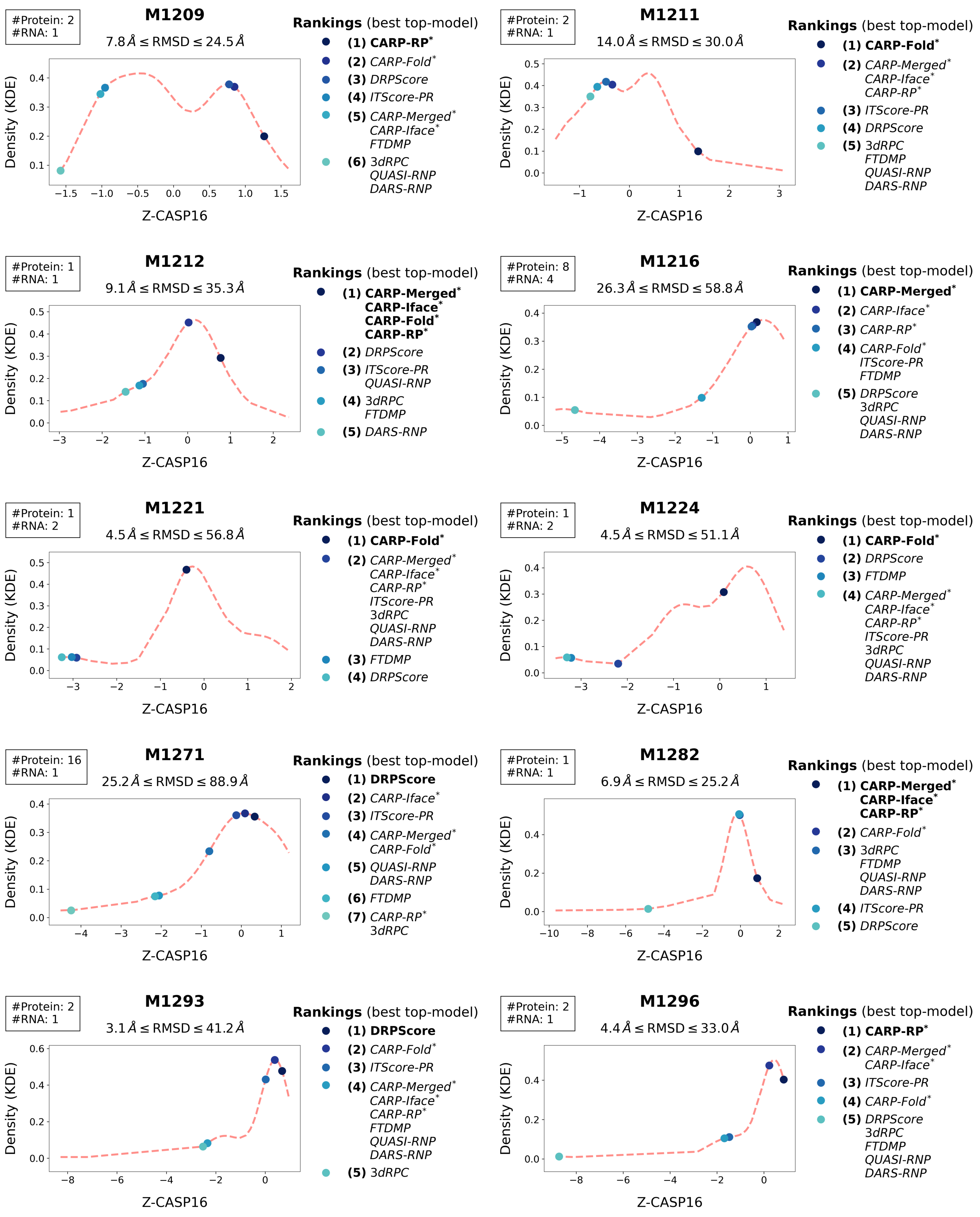

S.2.2 Blind-test target filtering

When comparing CARP against other external tools, for fair comparison, we filtered PDB ID's in the blind-test (total of 56 targets) that were similar to PDB ID's in the respective tools training set. Specifically, for 3dRPC [5], DRPScore [24], ITScore-PR [22], DARS-RNP [27], and QUASI-RNP [27], we fetched their respective training PDB IDs and filtered any blind-test PDB ID that had any protein-sequence (chain) or RNA-sequence (chain) with ≥ 0.3 or ≥ 0.8 sequence similarity, respectively, with the given training set using MMseqs2 [28]. We also filtered any blind-test PDB ID that had common RNAFam [29] clan/s with the given training set. This left a total of 27, 22, 17, and 16 targets for comparison with 3dRPC, DARS-RNP and QUASI-RNP, DRPScore, and ITScore-PR, respectively. For comparison with FTDMP, we were unable to find their respective training PDB IDs, thus for fair-comparison we used only the PDB IDs that were both in ours and their released blind-test, leaving a total of 26 targets.

S.2.3 AlphaFold3 results

S.2.3.1 Comparison with ipTM and pTM

To calculate the predicted TM and predicted interface TM score from AlphaFold3 [30], we used their predicted alignment error ($PAE \in \mathbb{R}^{N\times N}$) matrix output.

$$pTM = \max_{i\in[1,\ldots,N]} \left[\frac{1}{N} \sum_{j=1}^{N} \left(\frac{1}{1+\left(\frac{PAE[i,j]}{d_0(N)}\right)^2} \right) \right]$$

$$ipTM = \max_{i\in[1,\ldots,N]} \left[\frac{1}{N_{\text{inter}}} \sum_{\text{chain}(j)\neq\text{chain}(i)} \left(\frac{1}{1+\left(\frac{PAE[i,j]}{d_0(N)}\right)^2} \right) \right]$$

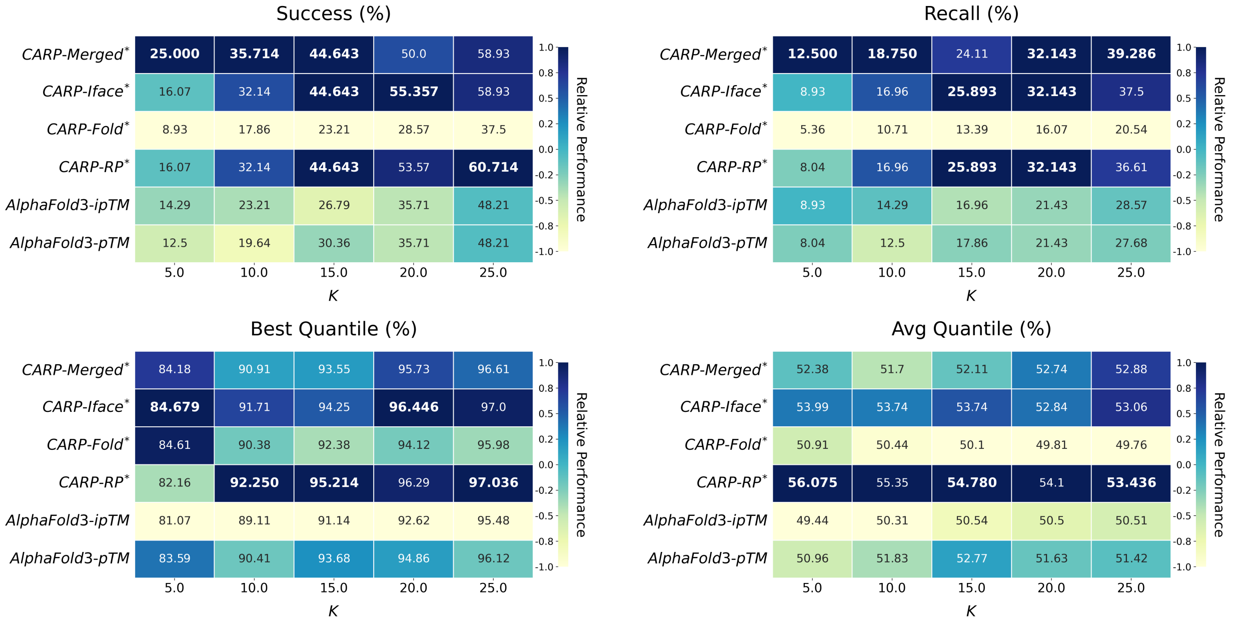

Figure S.11: Comparison results against AlphaFold3 Quality Estimates (all 56 targets) in terms of Success (A) and mean values for Recall (B), Best Quantile (C) and Average Quantile (D) with respect to iRMSD for the top-k selected decoys. The color indicates the relative performance of a method (min-max scaled). Darker colors indicate stronger performance.

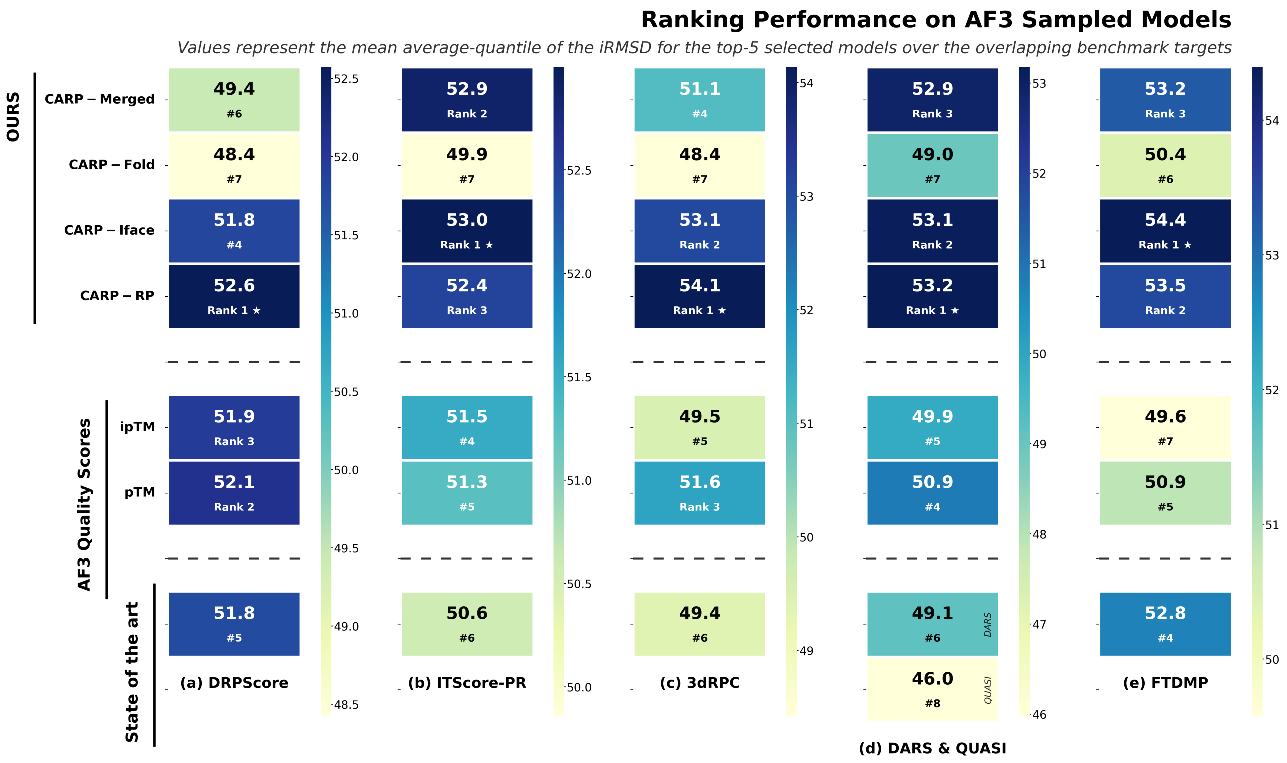

Figure S.12: AlphaFold3 blind-test (overlapping targets per-tool) results for Average Quantile with respect to iRMSD for the top-5 selected models. The color indicates the relative performance of a method (min-max scaled). Darker colors indicate stronger performance. Each column represents a separate blind test where only non-overlapping targets for the respective comparison method are used.

S.2.3.2 Comparison with other tools for Average Quantile, Best Quantile, Success, and Recall

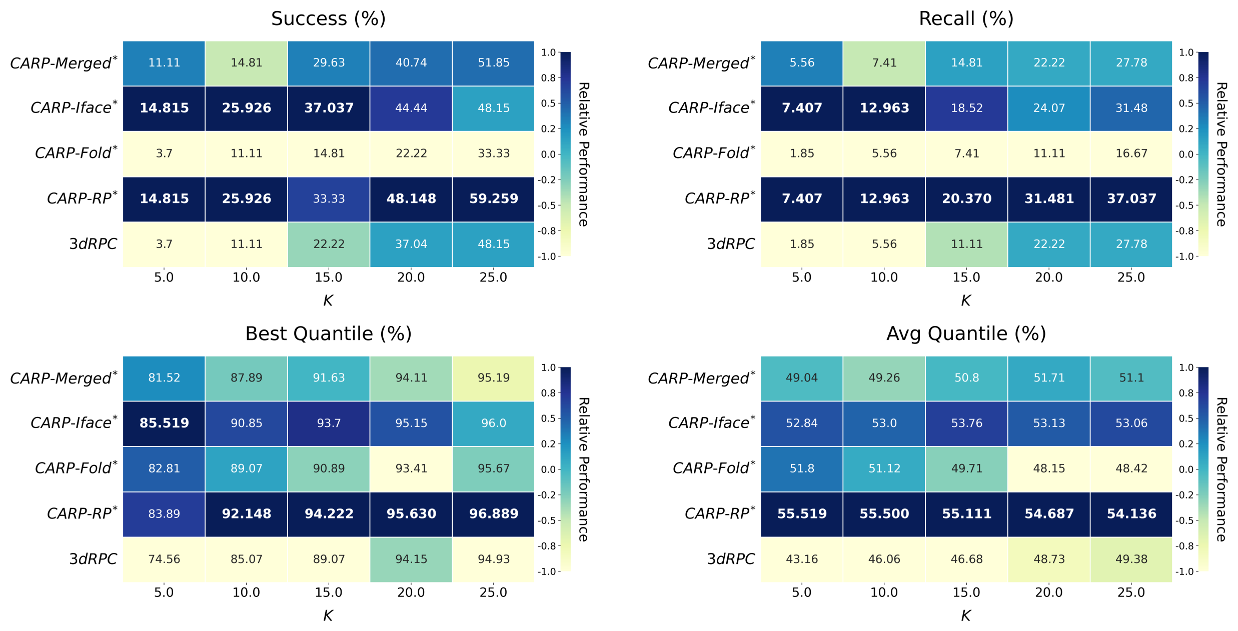

Figure S.13: Comparison results against 3dRPC Quality Estimates (27 targets) on the AlphaFold3 blind-test in terms of Success (A) and mean values for Recall (B), Best Quantile (C) and Average Quantile (D) with respect to iRMSD for the top-k selected decoys. The color indicates the relative performance of a method (min-max scaled). Darker colors indicate stronger performance.

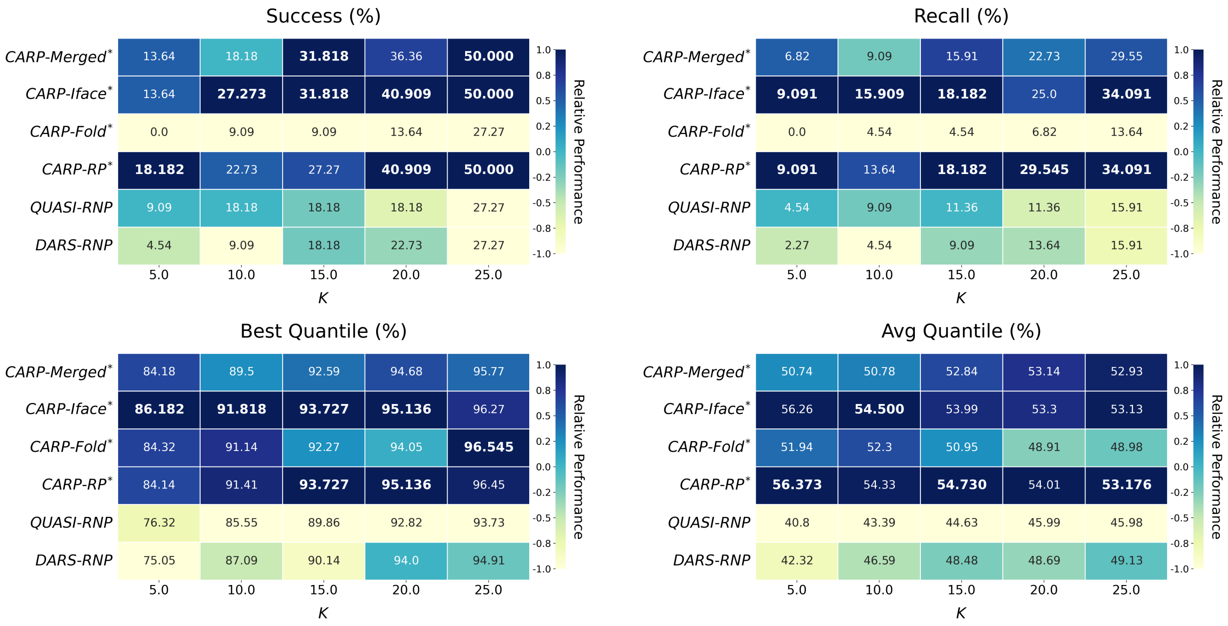

Figure S.14: Comparison results against DARS-RNP and QUASI-RNP Quality Estimates (22 targets) on the AlphaFold3 blind-test in terms of Success (A) and mean values for Recall (B), Best Quantile (C) and Average Quantile (D) with respect to iRMSD for the top-k selected decoys. The color indicates the relative performance of a method (min-max scaled). Darker colors indicate stronger performance.

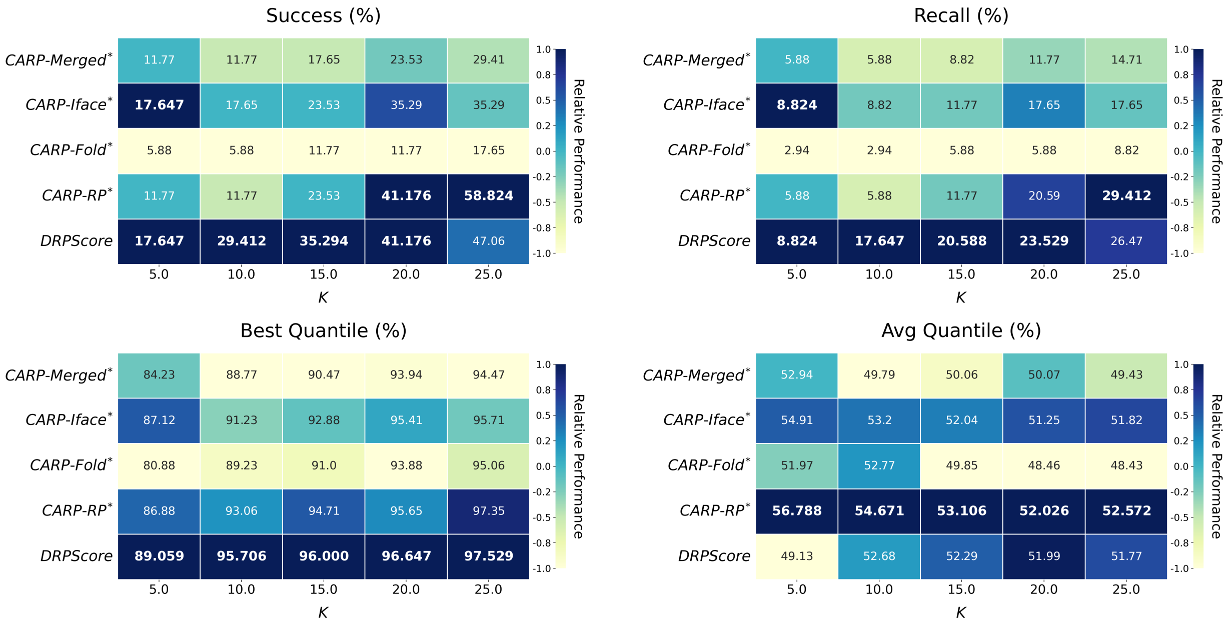

Figure S.15: Comparison results against DRPScore Quality Estimates (17 targets) on the AlphaFold3 blind-test in terms of Success (A) and mean values for Recall (B), Best Quantile (C) and Average Quantile (D) with respect to iRMSD for the top-k selected decoys. The color indicates the relative performance of a method (min-max scaled). Darker colors indicate stronger performance.

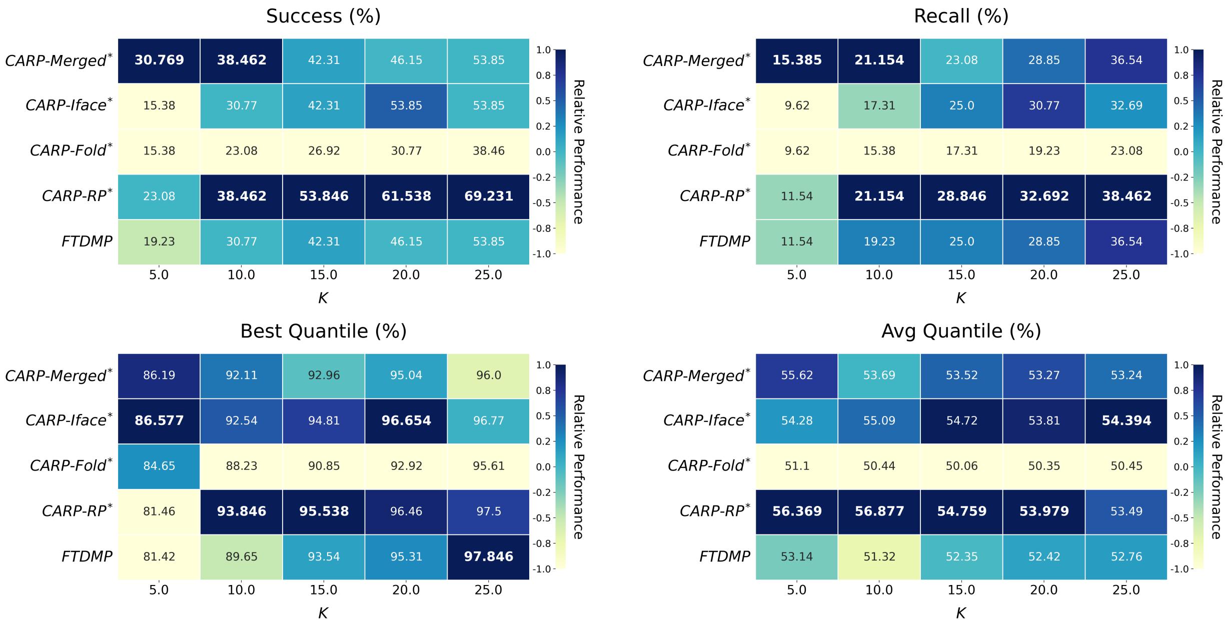

Figure S.16: Comparison results against FTDMP Quality Estimates (26 targets) on the AlphaFold3 blind-test in terms of Success (A) and mean values for Recall (B), Best Quantile (C) and Average Quantile (D) with respect to iRMSD for the top-k selected decoys. The color indicates the relative performance of a method (min-max scaled). Darker colors indicate stronger performance.

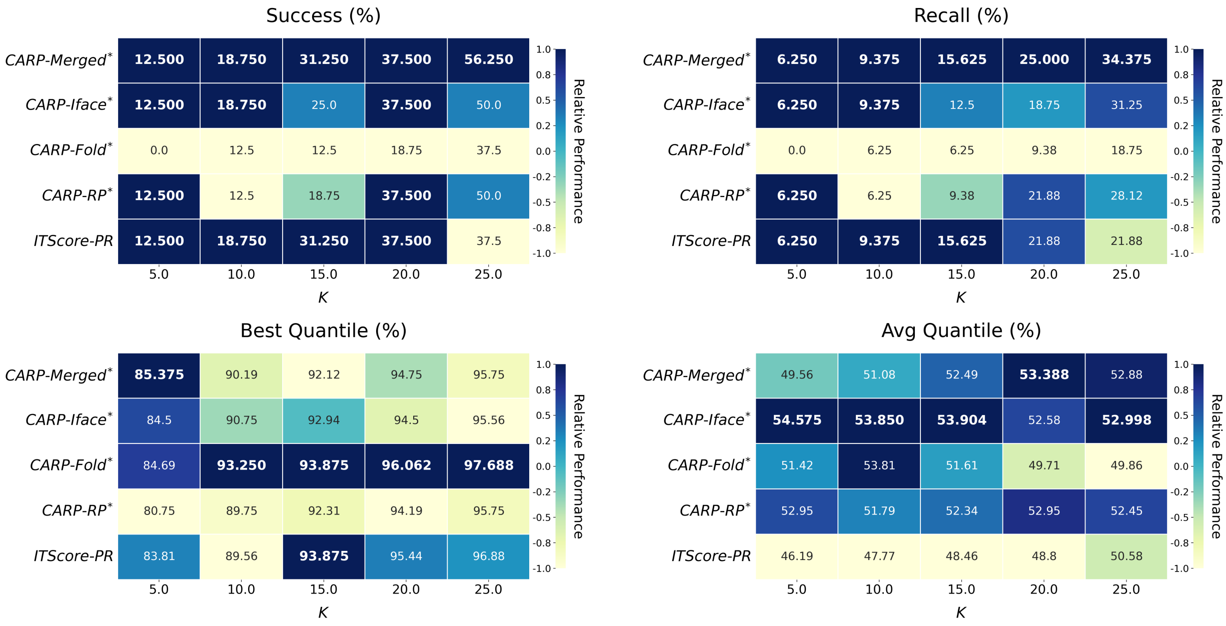

Figure S.17: Comparison results against ITScore-PR Quality Estimates (16 targets) on the AlphaFold3 blind-test in terms of Success (A) and mean values for Recall (B), Best Quantile (C) and Average Quantile (D) with respect to iRMSD for the top-k selected decoys. The color indicates the relative performance of a method (min-max scaled). Darker colors indicate stronger performance.

S.2.3.3 Per-complex kernel density estimation and rankings

For the subsequent figures related to AlphaFold3 KDE, the iRMSD corresponds to the interface fitted iRMSD described in Section S.1.6.4.

Figure S.18: Density (KDE) and predictor rankings per-target for iRMSD on the AlphaFold3 blind-test non-dimer targets (1-10).

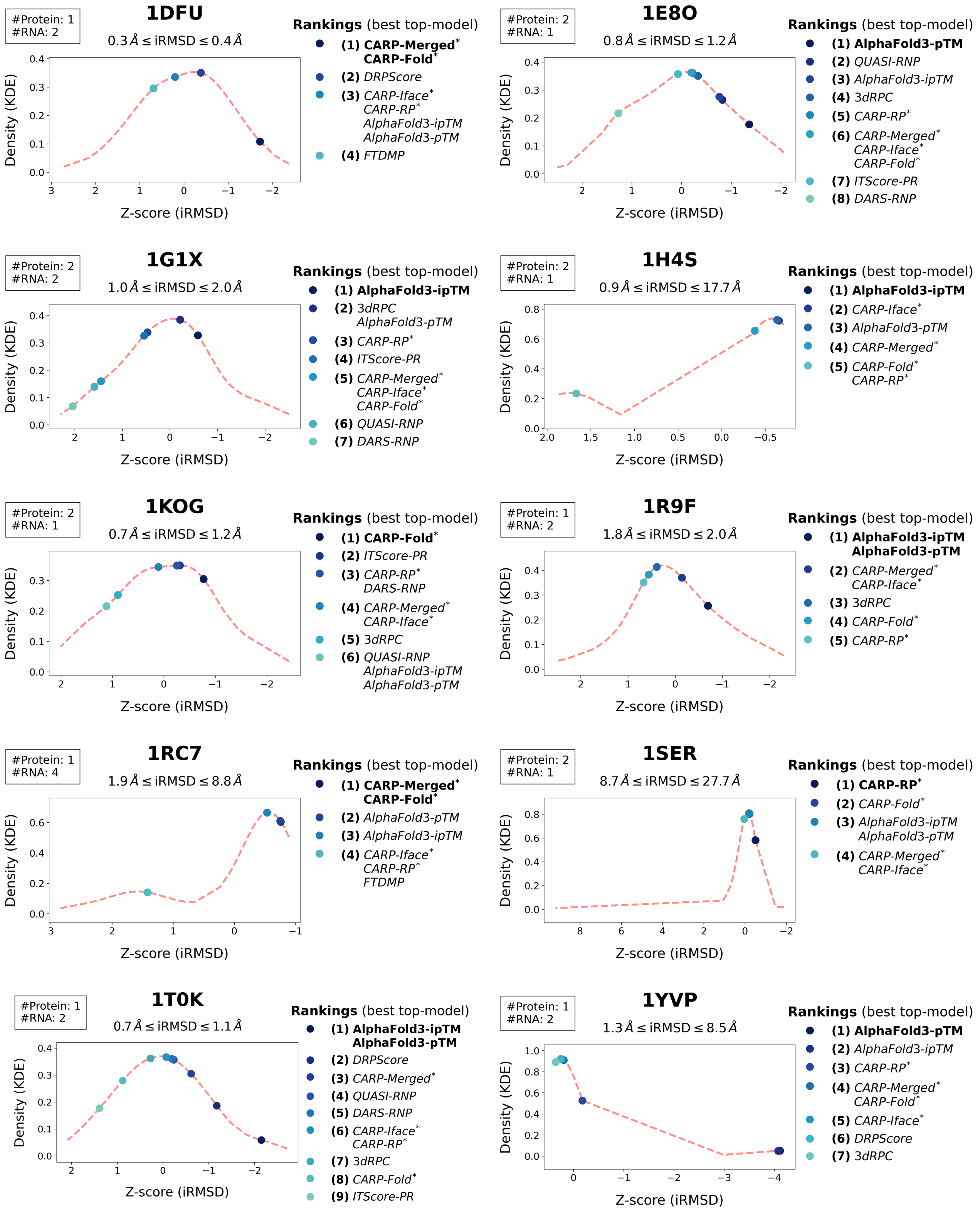

Figure S.19: Density (KDE) and predictor rankings per-target for iRMSD on the AlphaFold3 blind-test non-dimer targets (10-18).

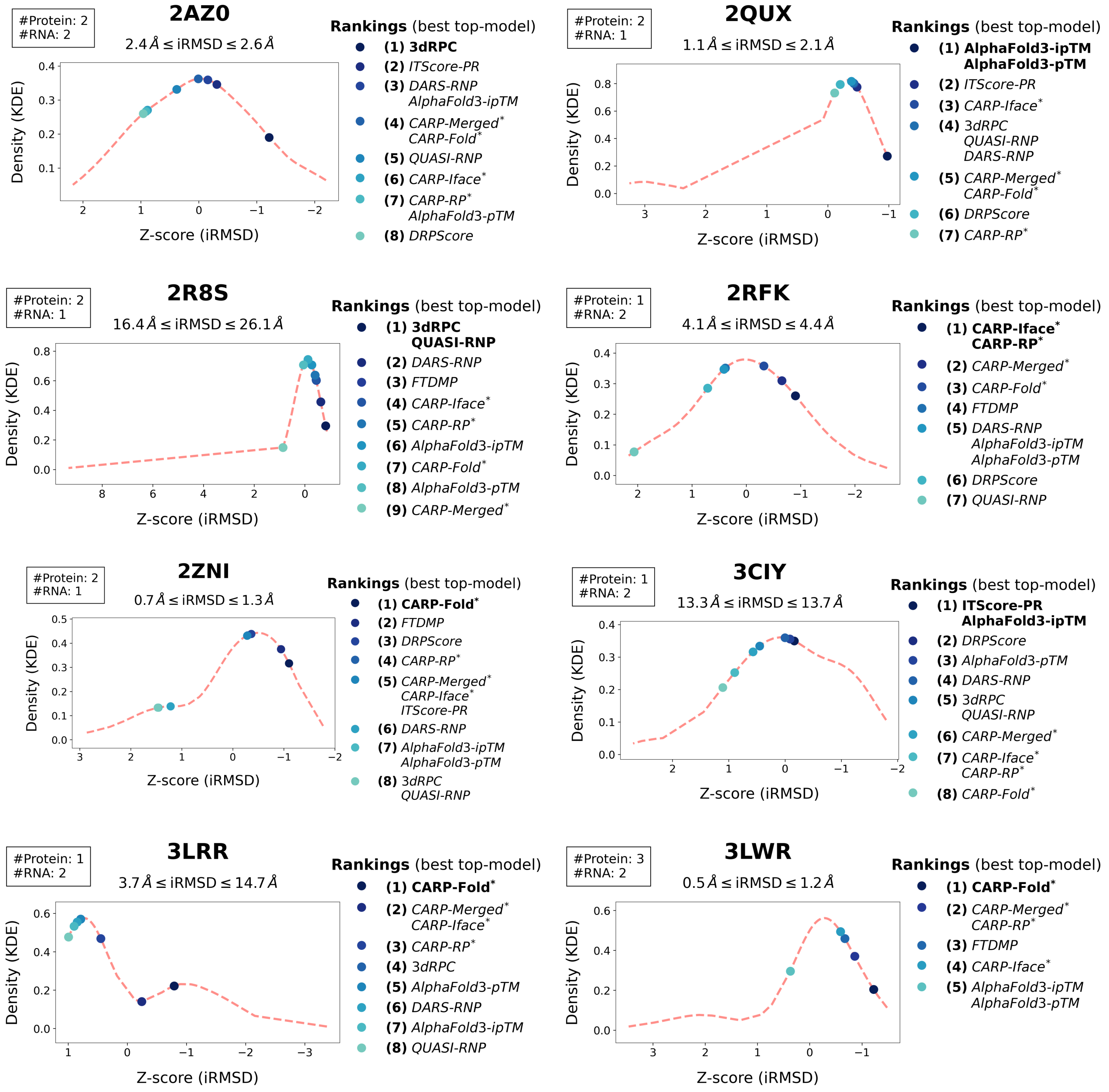

Figure S.20: Density (KDE) and predictor rankings per-target for iRMSD on the AlphaFold3 blind-test dimer targets (1-10).

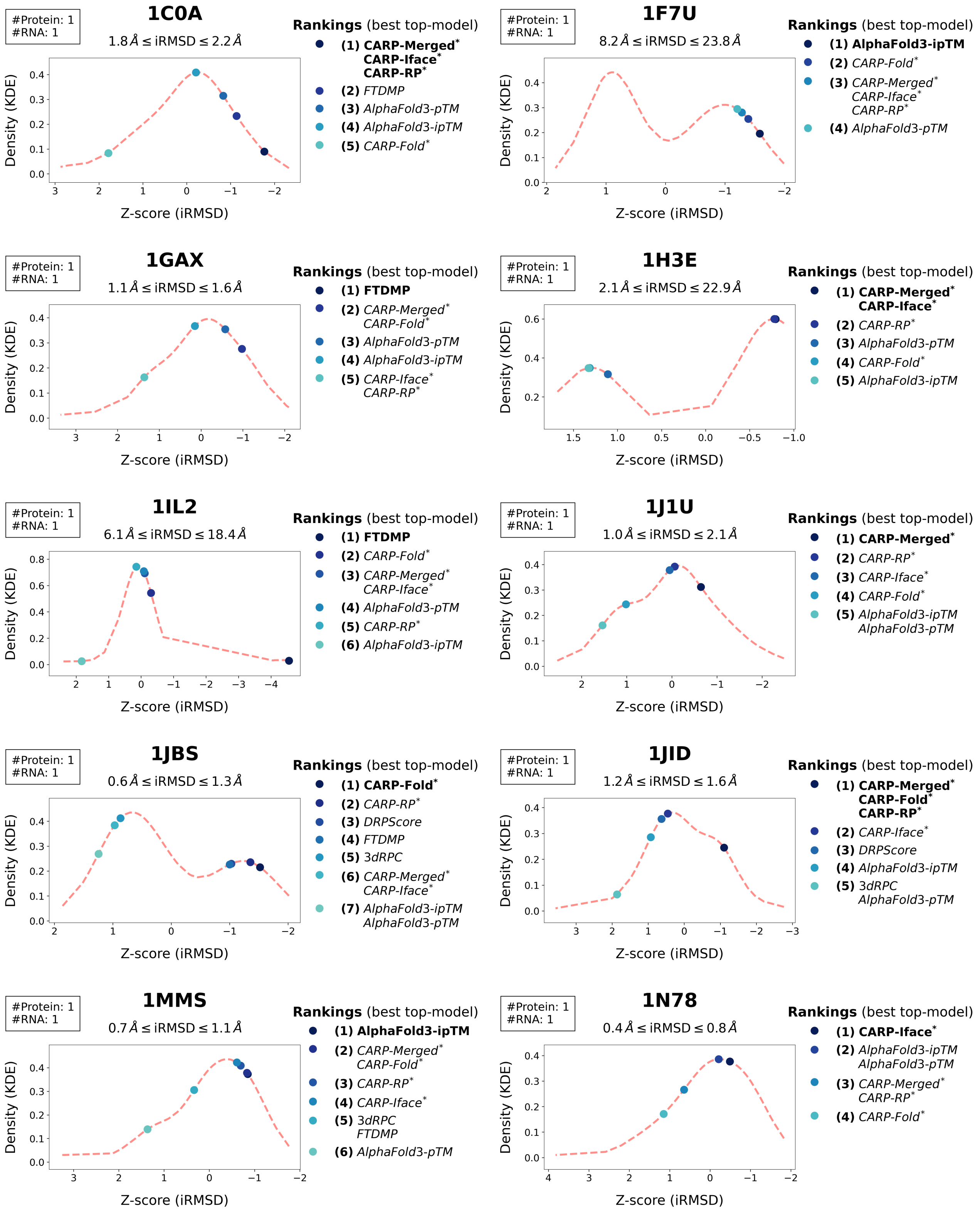

Figure S.21: Density (KDE) and predictor rankings per-target for iRMSD on the AlphaFold3 blind-test dimer targets (10-20).

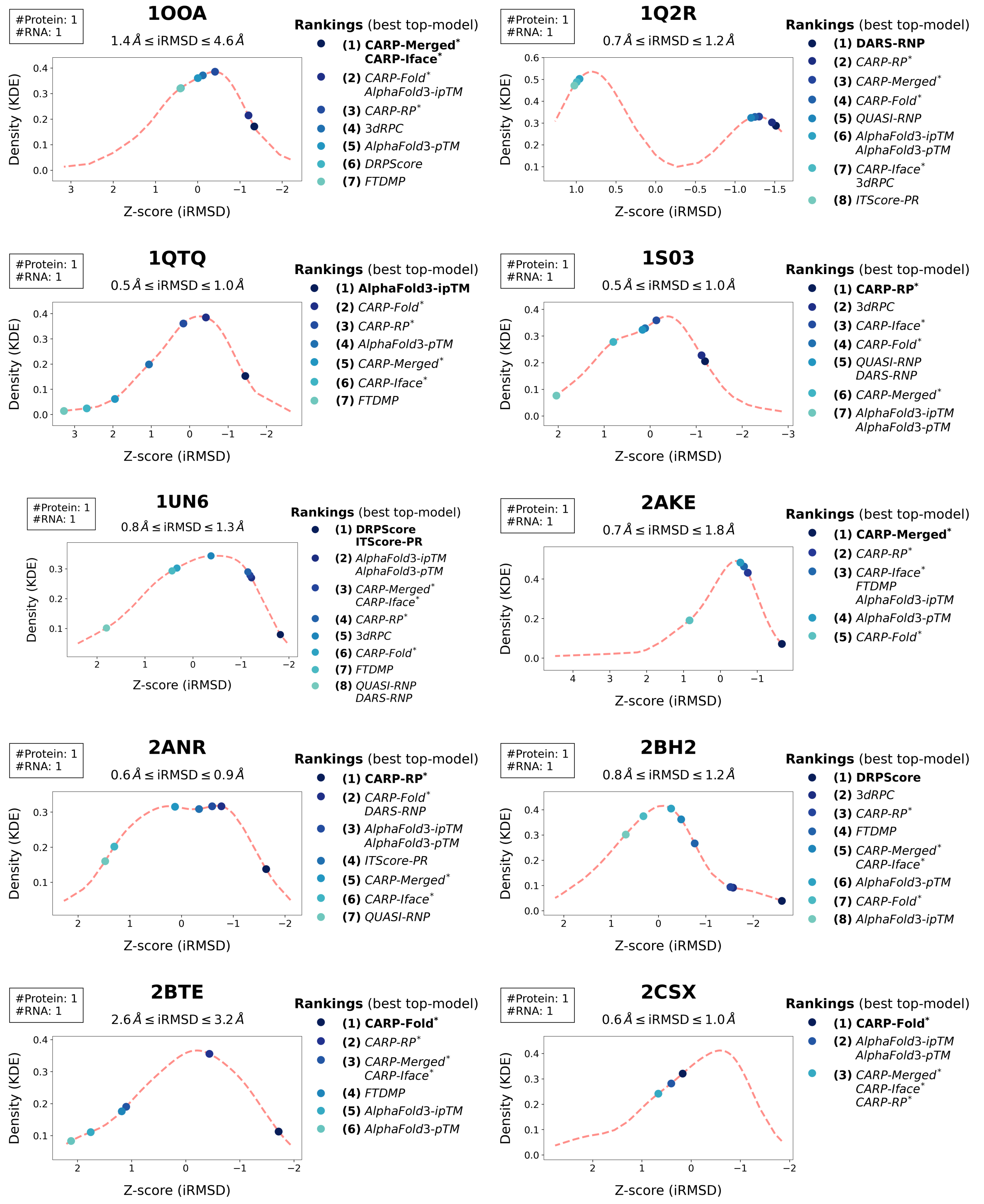

Figure S.22: Density (KDE) and predictor rankings per-target for iRMSD on the AlphaFold3 blind-test dimer targets (20-30).

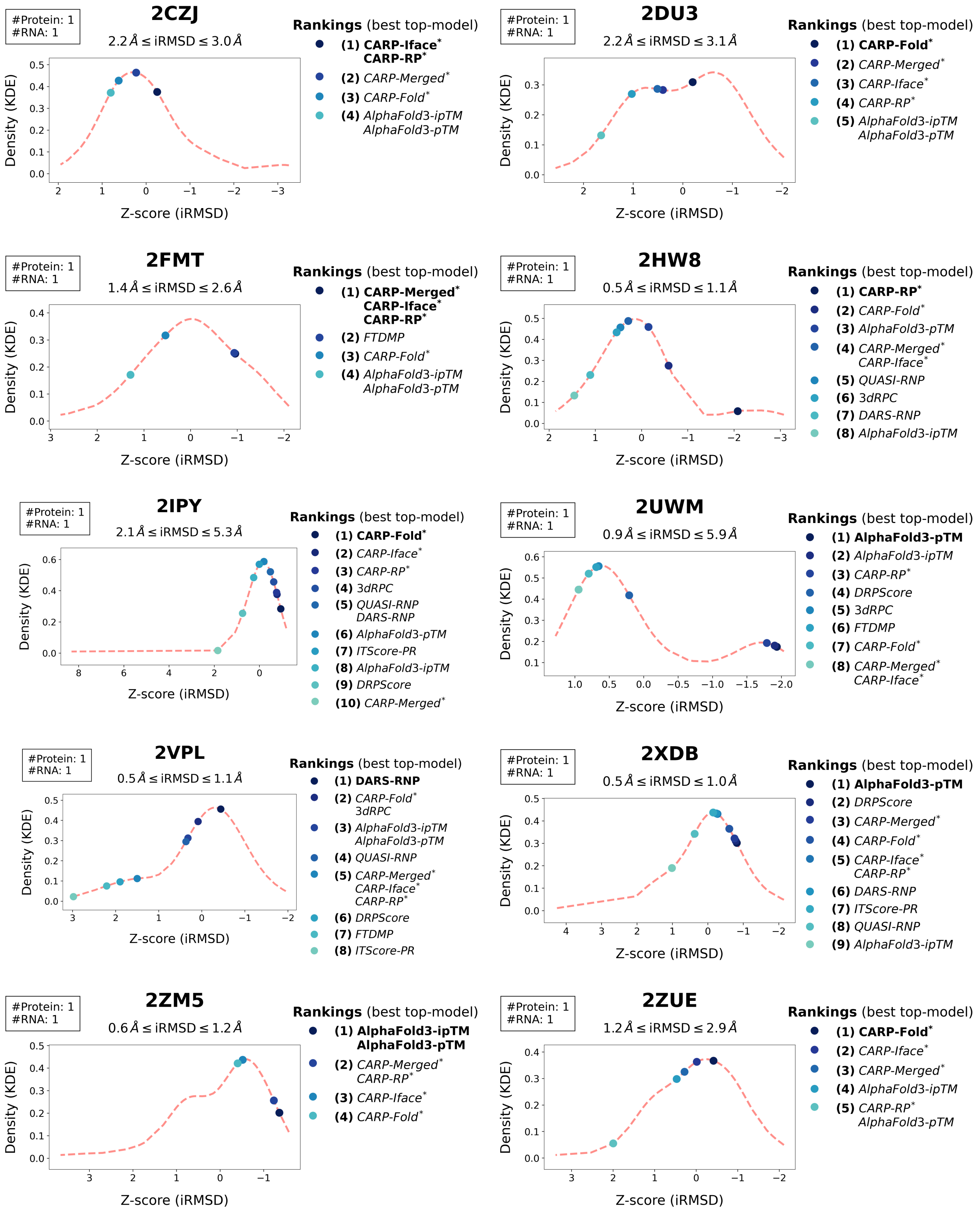

Figure S.23: Density (KDE) and predictor rankings per-target for iRMSD on the AlphaFold3 blind-test dimer targets (30-38).

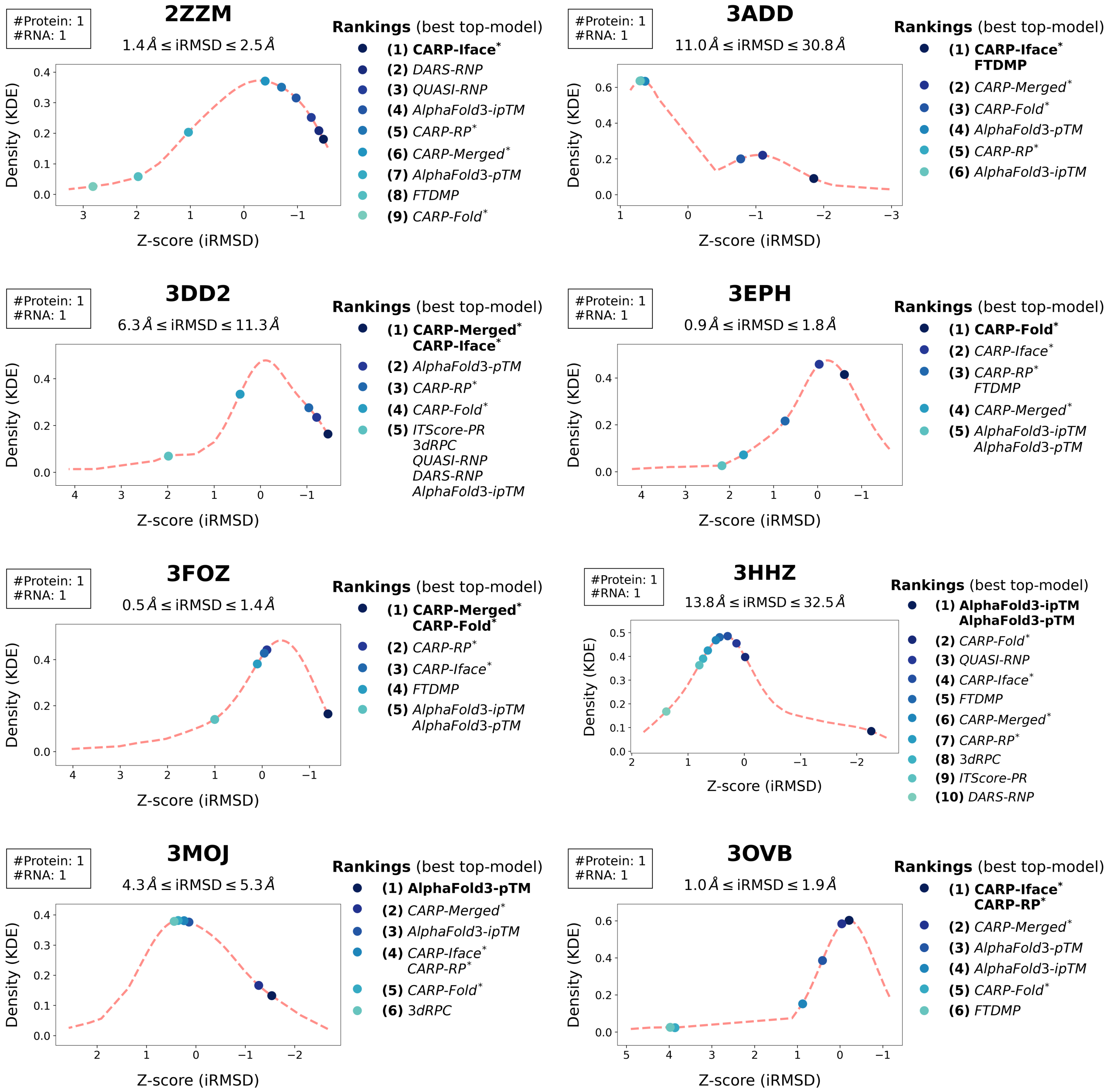

S.2.4 Docking results

S.2.4.1 Average Quantile, Best Quantile, Recall, and Success

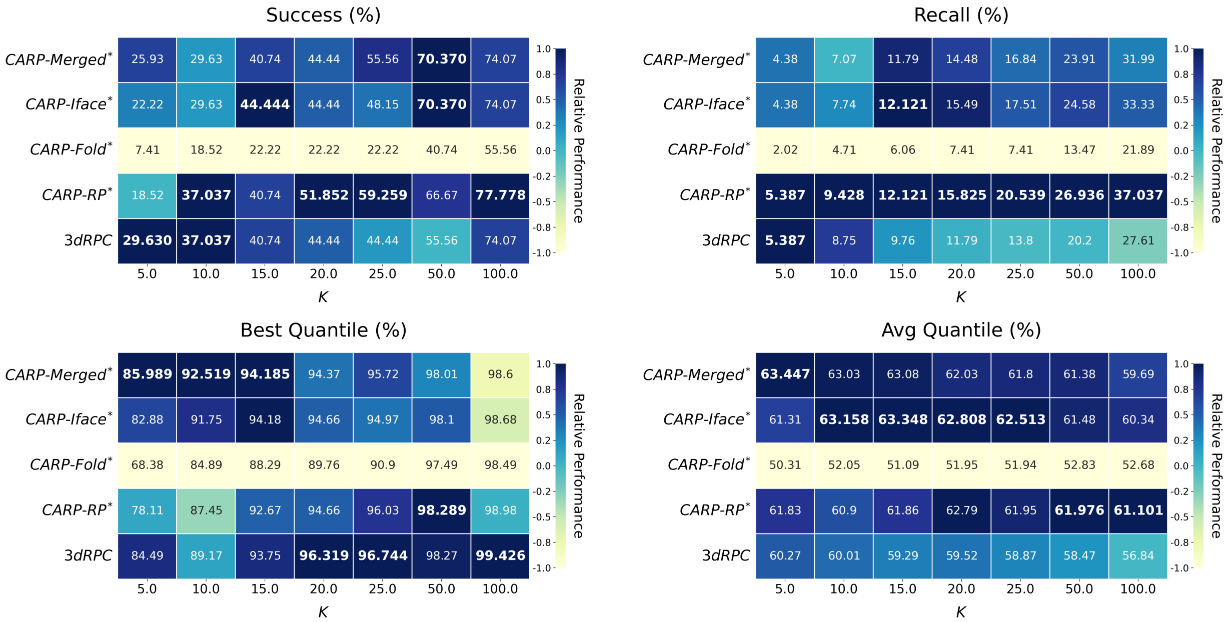

Figure S.24: Comparison results against 3dRPC Quality Estimates (27 targets) on the docking blind-test in terms of Success (A) and mean values for Recall (B), Best Quantile (C) and Average Quantile (D) with respect to iRMSD for the top-k selected decoys. The color indicates the relative performance of a method (min-max scaled). Darker colors indicate stronger performance.

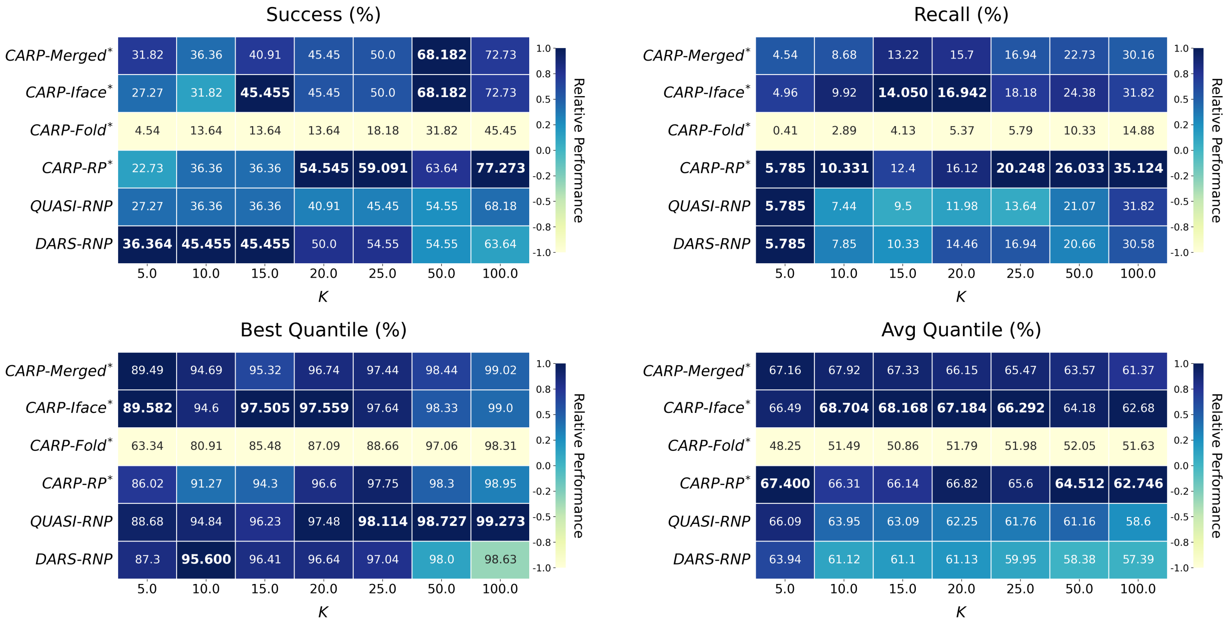

Figure S.25: Comparison results against DARS-RNP and QUASI-RNP Quality Estimates (22 targets) on the docking blind-test in terms of Success (A) and mean values for Recall (B), Best Quantile (C) and Average Quantile (D) with respect to iRMSD for the top-k selected decoys. The color indicates the relative performance of a method (min-max scaled). Darker colors indicate stronger performance.

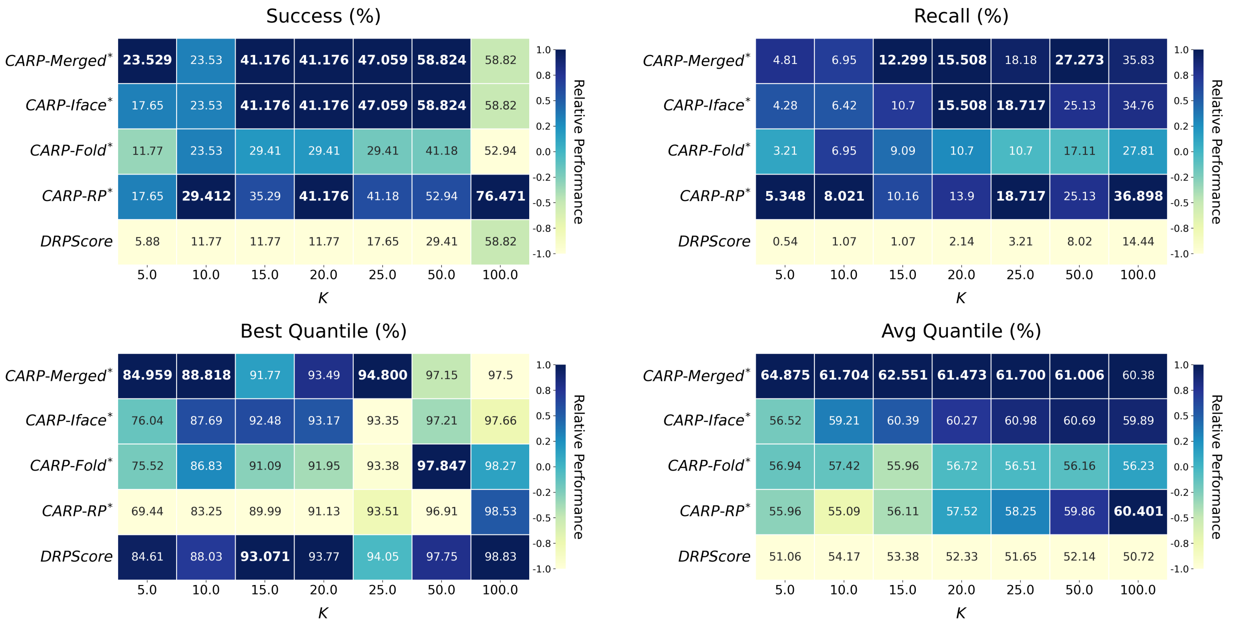

Figure S.26: Comparison results against DRPScore Quality Estimates (17 targets) on the docking blind-test in terms of Success (A) and mean values for Recall (B), Best Quantile (C) and Average Quantile (D) with respect to iRMSD for the top-k selected decoys. The color indicates the relative performance of a method (min-max scaled). Darker colors indicate stronger performance.

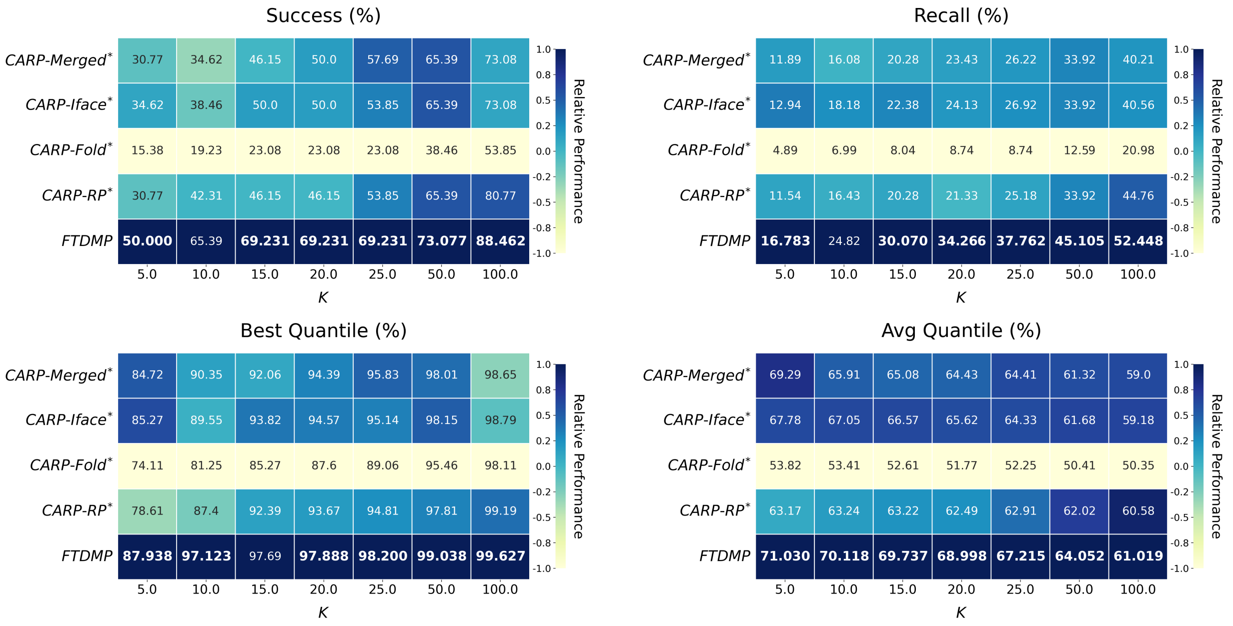

Figure S.27: Comparison results against FTDMP Quality Estimates (26 targets) on the docking blind-test in terms of Success (A) and mean values for Recall (B), Best Quantile (C) and Average Quantile (D) with respect to iRMSD for the top-k selected decoys. The color indicates the relative performance of a method (min-max scaled). Darker colors indicate stronger performance.

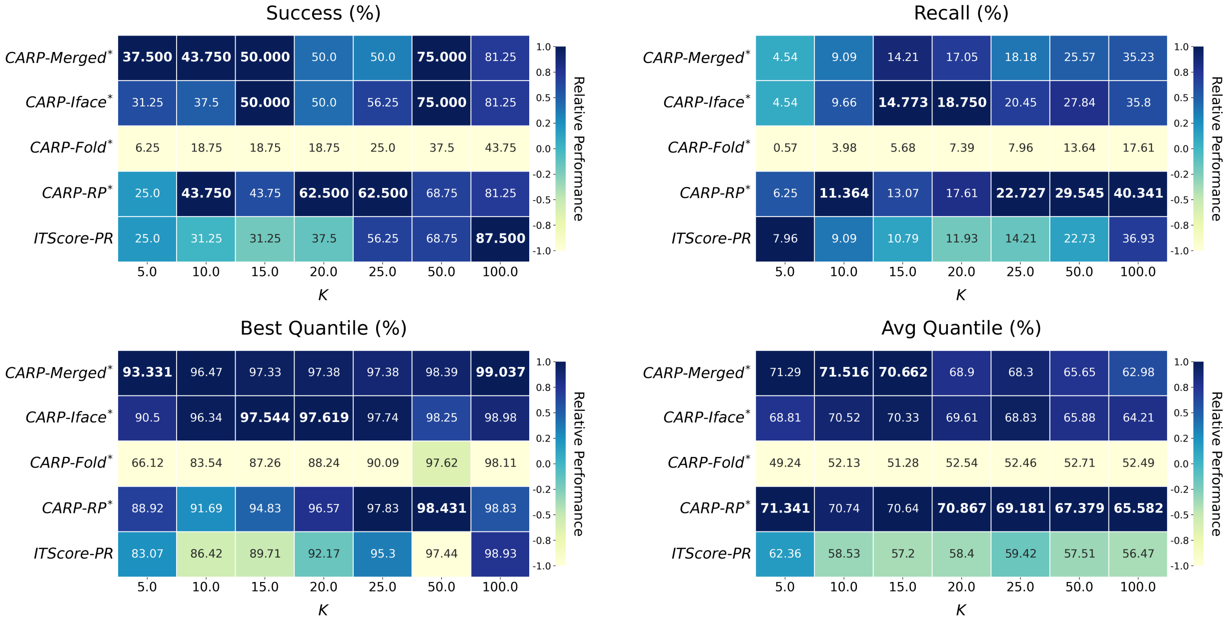

Figure S.28: Comparison results against ITScore-PR Quality Estimates (16 targets) on the docking blind-test in terms of Success (A) and mean values for Recall (B), Best Quantile (C) and Average Quantile (D) with respect to iRMSD for the top-k selected decoys. The color indicates the relative performance of a method (min-max scaled). Darker colors indicate stronger performance.

S.2.4.2 Per-complex kernel density estimation and rankings

For the subsequent figures related to docking KDE, the iRMSD corresponds to the protein fitted iRMSD described in Section S.1.6.4.

Figure S.29: Density (KDE) and predictor rankings per-target for iRMSD on the docking blind-test non-dimer targets (1-10).

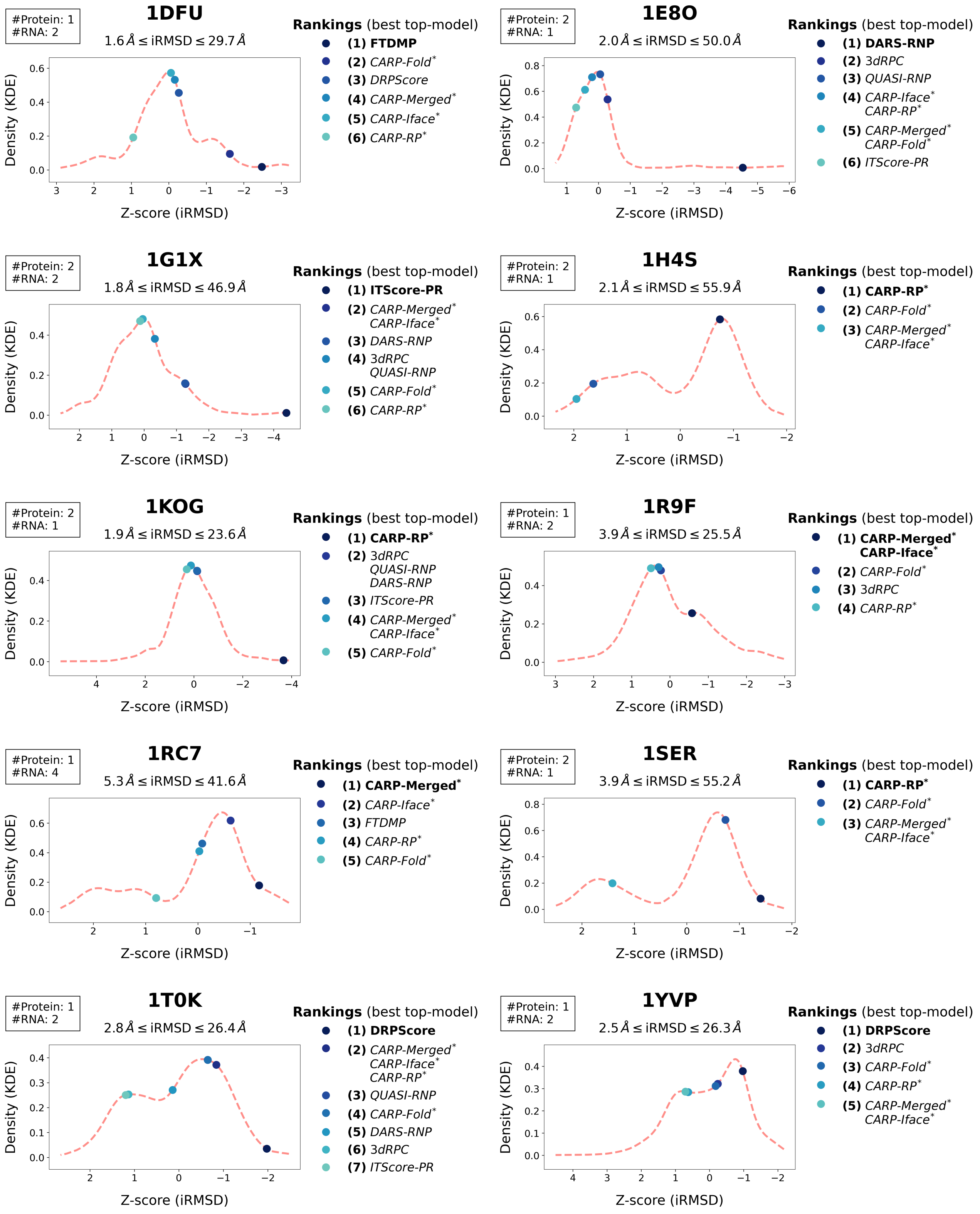

Figure S.30: Density (KDE) and predictor rankings per-target for iRMSD on the docking blind-test non-dimer targets (10-18).

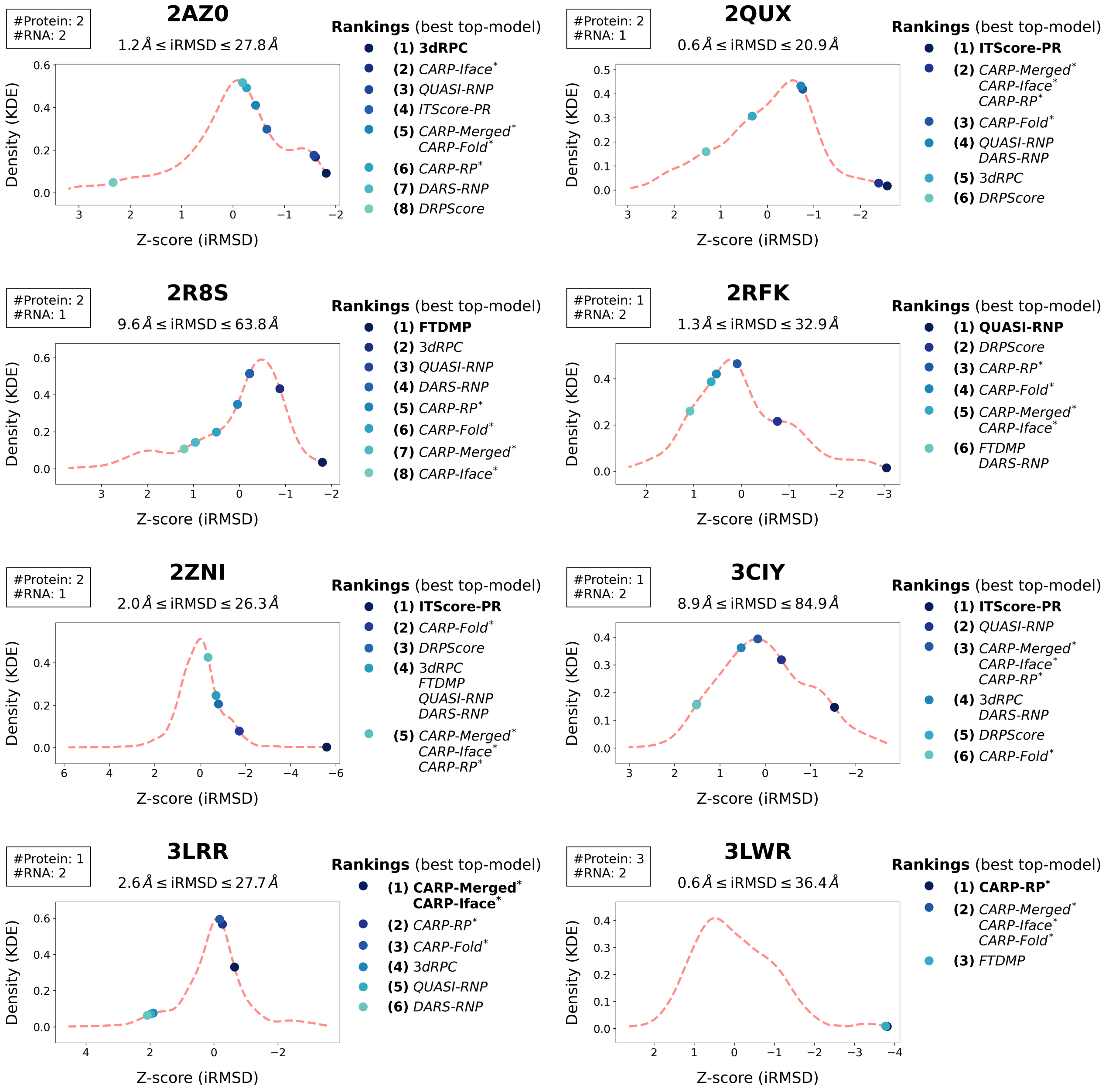

Figure S.31: Density (KDE) and predictor rankings per-target for iRMSD on the docking blind-test dimer targets (1-10).

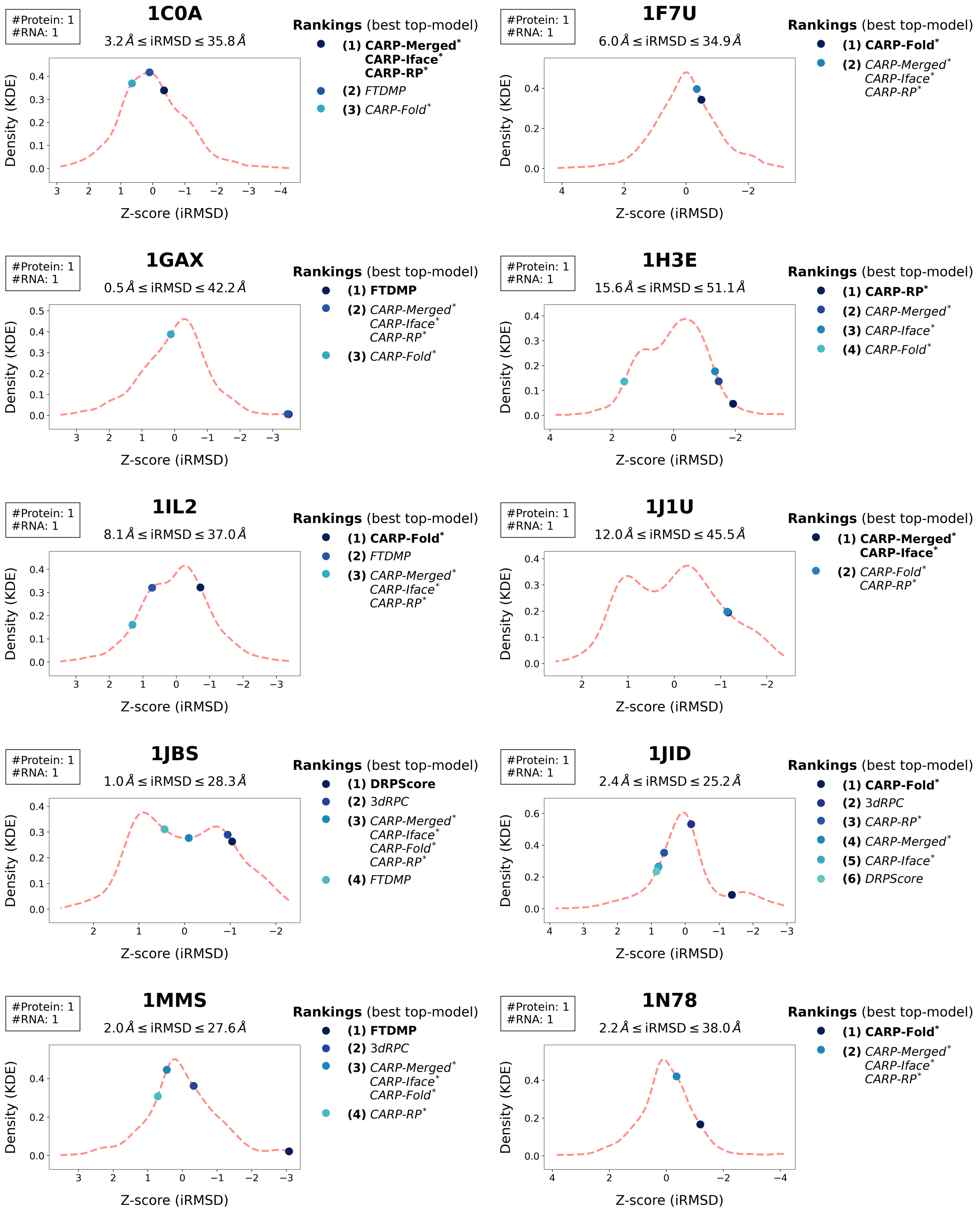

Figure S.32: Density (KDE) and predictor rankings per-target for iRMSD on the docking blind-test dimer targets (10-20).

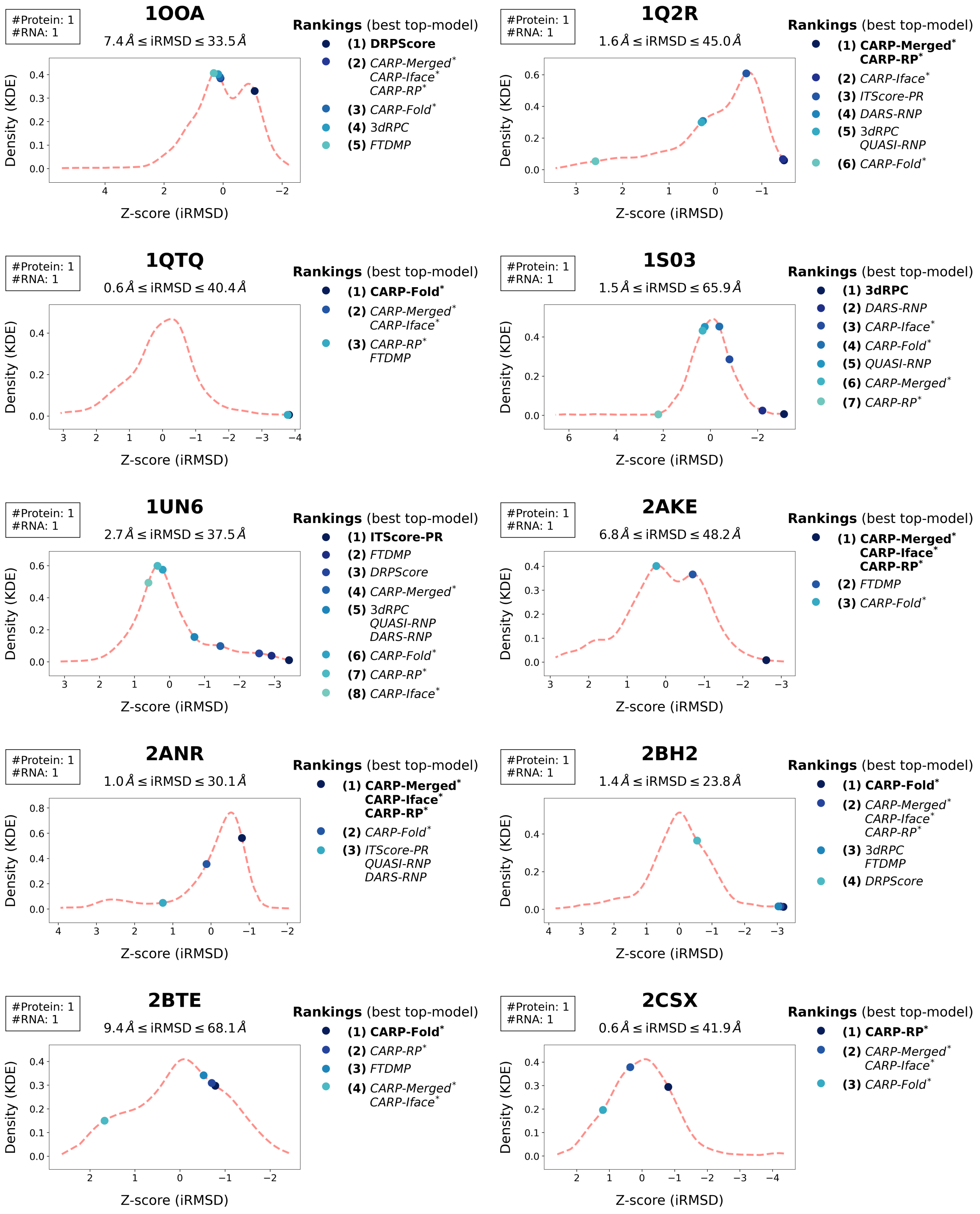

Figure S.33: Density (KDE) and predictor rankings per-target for iRMSD on the docking blind-test dimer targets (20-30).

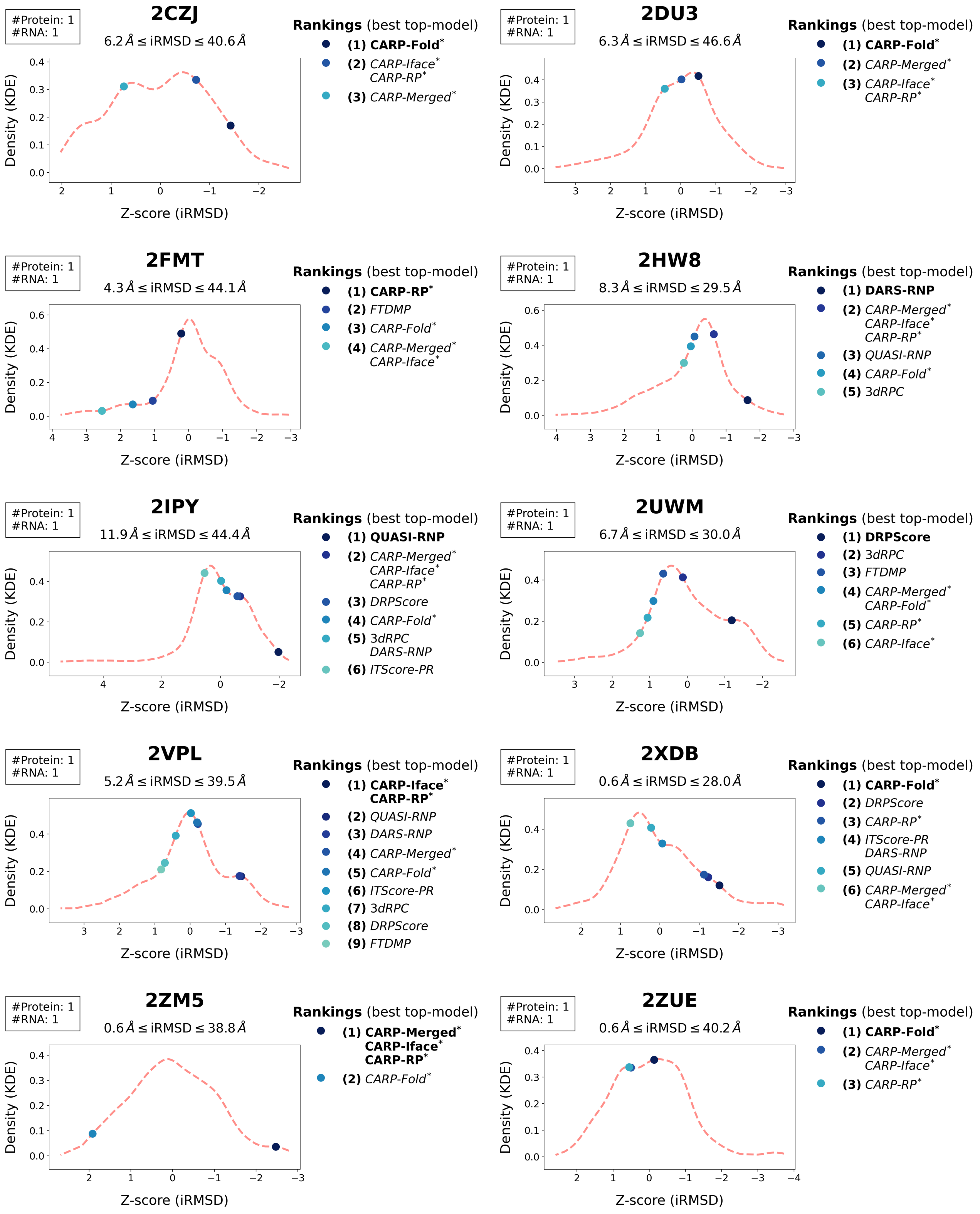

Figure S.34: Density (KDE) and predictor rankings per-target for iRMSD on the docking blind-test dimer targets (30-38).

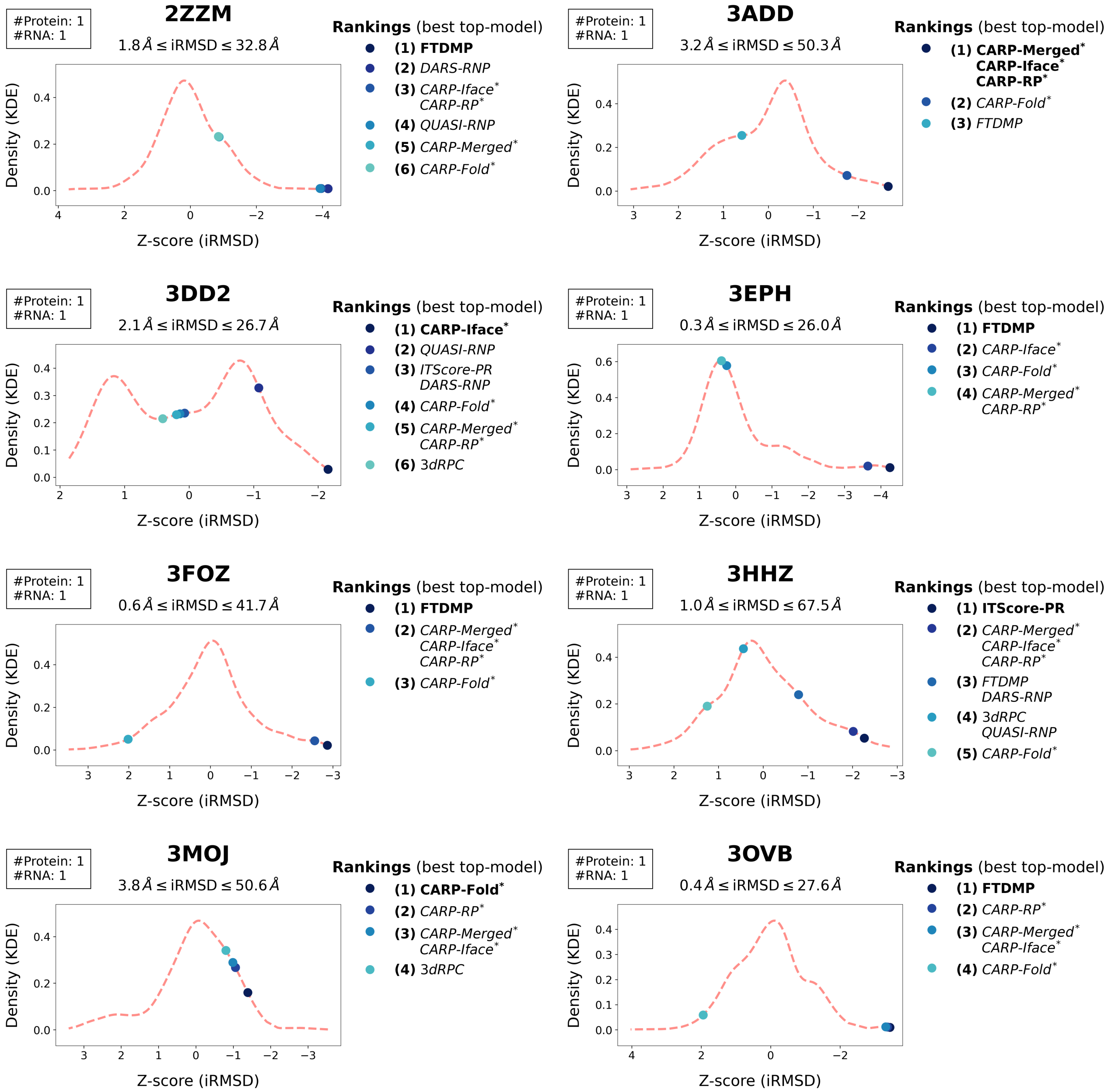

S.2.5 Feature analysis

For each graph in the respective dataset, we applied random within-graph (polymer-specific) permutations on a feature's respective channels (8 shuffles for each feature per-model in the blind-test dataset, 32 shuffles for each feature per-model in the CASP dataset), and calculated the absolute change in prediction for each of CARP's global quality scores. We then, converted these change in quality scores $|\Delta|$ into within-model quantiles, $Q(|\Delta|)$, over all of the features. Note that feature importance for graph-neural networks is an active area of research, and for simplicity we decided to use permutation based feature sensitivity, to avoid out of distribution features impacting the analysis.

Figure S.35: Feature sensitivity results for CASP16. Sensitivity is defined as the within-model quantile of the change in prediction under random feature-specific permutations. Box plots are labeled based upon the respective polymer they are derived/involved in.

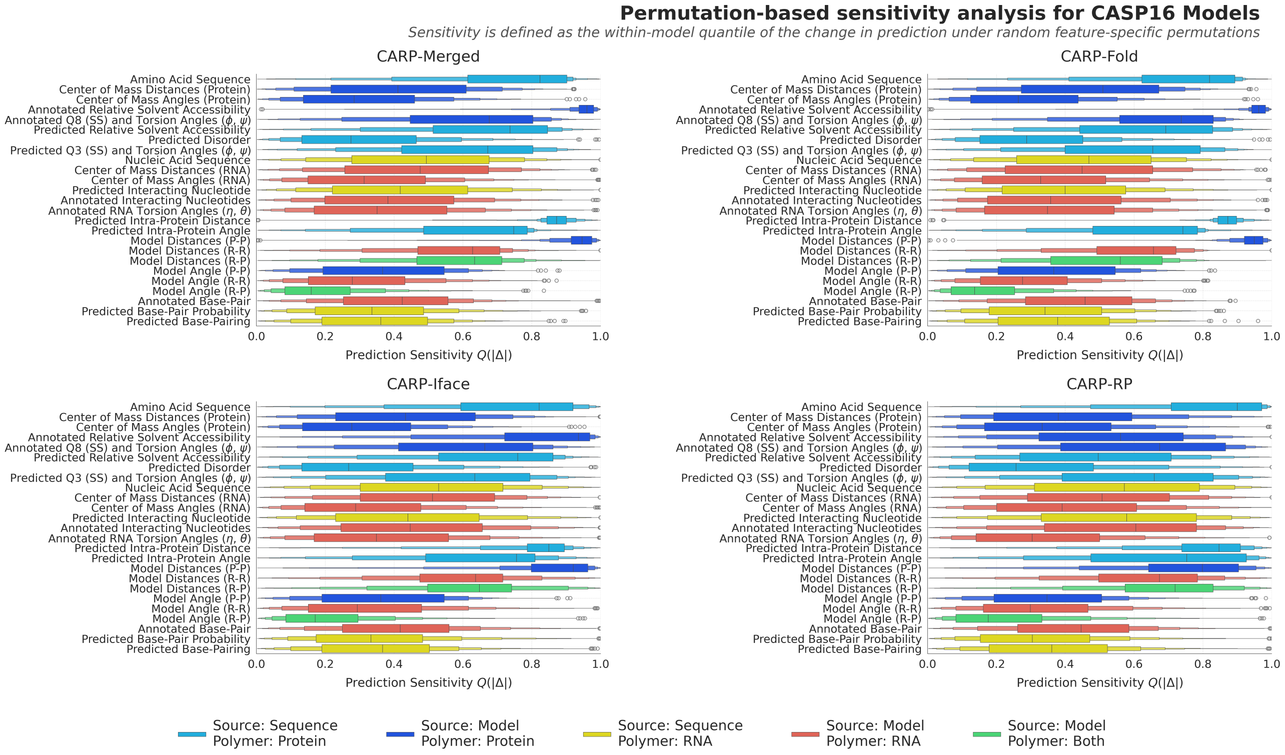

Figure S.36: Feature sensitivity results for the docking blind-test. Sensitivity is defined as the within-model quantile of the change in prediction under random feature-specific permutations. Box plots are labeled based upon the respective polymer they are derived/involved in.

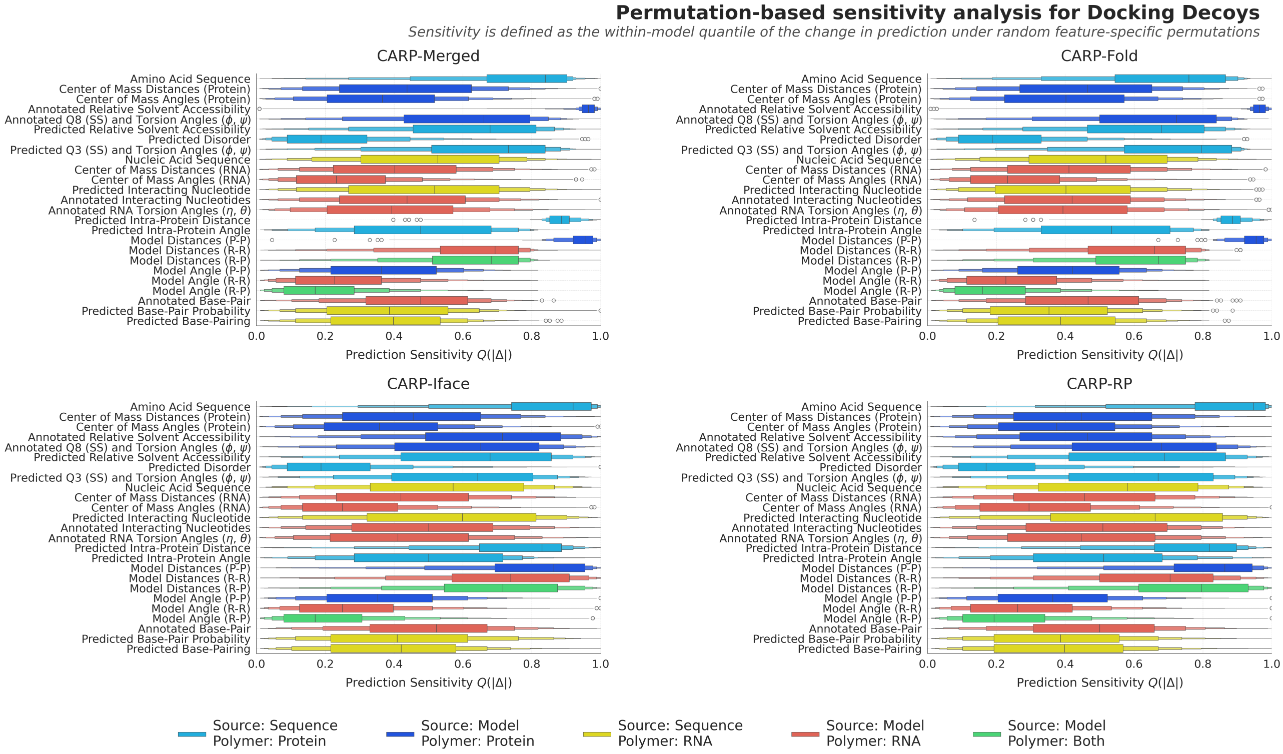

S.2.6 Docking perturbation analysis

The docking results and perturbed docking results are shown in Figures S.37 and S.38, respectively, for all 56 protein-RNA complexes. Emphasizing the effect of perturbations, we observe that CARP-RP's performance generally decreases, while CARP-Merged's performance increases. This provides insight into how structural perturbations can influence CARP-predicted scores during model selection, as they further affect overall fold configuration while restricting changes in the relative orientation of the chains. Therefore, these findings imply that the CARP-Merged score, which predicts global fold and interface quality, could be better suited for model selection under perturbed conditions.

Figure S.37: Results on the docking blind-test targets (all 56 targets) in terms of Success (A) and mean values for Recall (B), Best Quantile (C) and Average Quantile (D) with respect to iRMSD for the top-k selected decoys. The color indicates the relative performance of a method (min-max scaled). Darker colors indicate stronger performance.

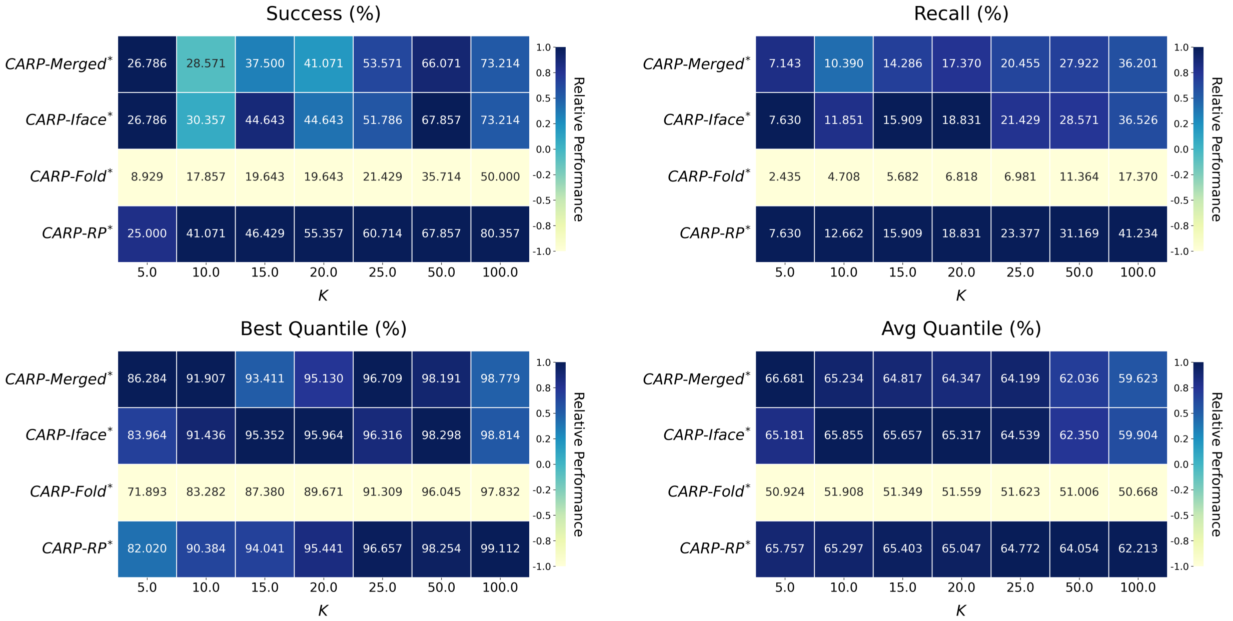

Figure S.38: Results on the perturbed docking blind-test targets (all 56 targets) in terms of Success (A) and mean values for Recall (B), Best Quantile (C) and Average Quantile (D) with respect to iRMSD for the top-k selected decoys. The color indicates the relative performance of a method (min-max scaled). Darker colors indicate stronger performance.

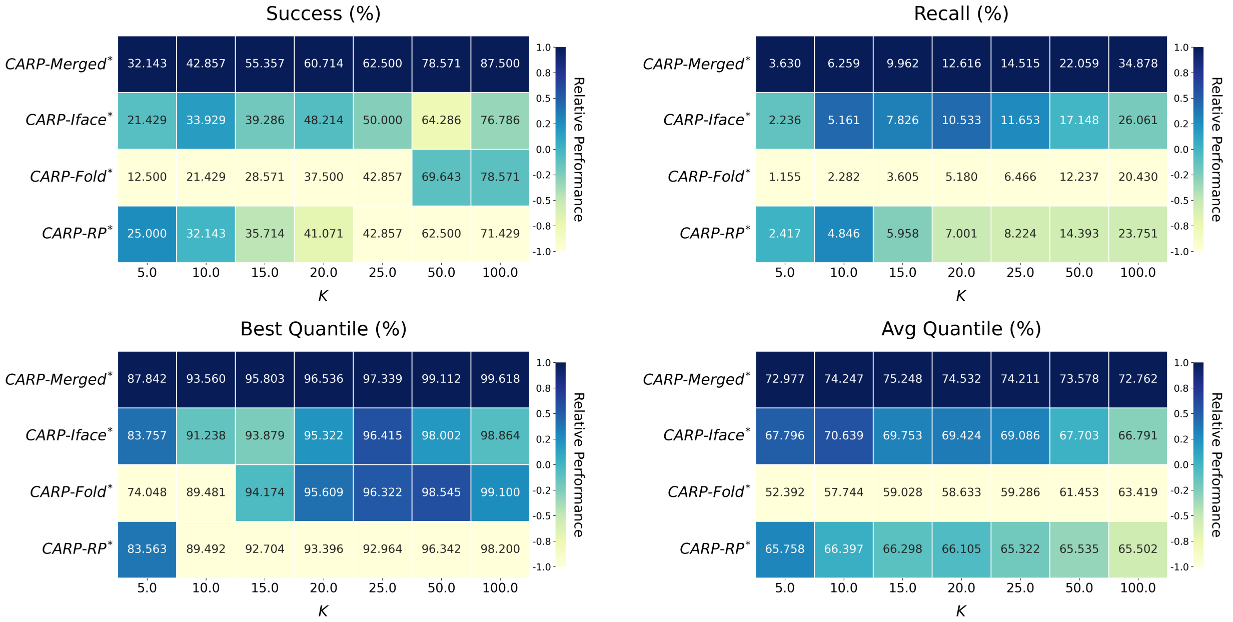

References

[1] A Paszke. Pytorch: An imperative style, high-performance deep learning library. *arXiv preprint arXiv:1912.01703*, 2019.

[2] Matthias Fey and Jan Eric Lenssen. Fast graph representation learning with PyTorch Geometric. In *ICLR Workshop on Representation Learning on Graphs and Manifolds*, 2019.

[3] Ilya Loshchilov and Frank Hutter. Decoupled weight decay regularization. *arXiv preprint arXiv:1711.05101*, 2017.

[4] Prem Seetharaman, Gordon Wichern, Bryan Pardo, and Jonathan Le Roux. Autoclip: Adaptive gradient clipping for source separation networks. In *2020 IEEE 30th International Workshop on Machine Learning for Signal Processing (MLSP)*, pages 1–6. IEEE, 2020.

[5] Haotian Li, Yangyu Huang, and Yi Xiao. A pair-conformation-dependent scoring function for evaluating 3d rna-protein complex structures. *PLoS One*, 12(3):e0174662, 2017.

[6] Sidhartha Chaudhury, Sergey Lyskov, and Jeffrey J Gray. Pyrosetta: a script-based interface for implementing molecular modeling algorithms using rosetta. *Bioinformatics*, 26(5):689–691, 2010.

[7] Marco Biasini, Tobias Schmidt, Stefan Bienert, Valerio Mariani, Gabriel Studer, Jürgen Haas, Niklaus Johner, Andreas Daniel Schenk, Ansgar Philippsen, and Torsten Schwede. Openstructure: an integrated software framework for computational structural biology. *Biological crystallography*, 69(5):701–709, 2013.

[8] Kengo Sato, Yuki Kato, Michiaki Hamada, Tatsuya Akutsu, and Kiyoshi Asai. Ipknot: fast and accurate prediction of rna secondary structures with pseudoknots using integer programming. *Bioinformatics*, 27(13):i85–i93, 2011.

[9] Magnus Haraldson Høie, Erik Nicolas Kiehl, Bent Petersen, Morten Nielsen, Ole Winther, Henrik Nielsen, Jeppe Hallgren, and Paolo Marcatili. Netsurfp-3.0: accurate and fast prediction of protein structural features by protein language models and deep learning. *Nucleic acids research*, 50(W1):W510–W515, 2022.

[10] Carlos M Duarte and Anna Marie Pyle. Stepping through an rna structure: a novel approach to conformational analysis. *Journal of molecular biology*, 284(5):1465–1478, 1998.

[11] Huanwang Yang, Fabrice Jossinet, Neocles Leontis, Li Chen, John Westbrook, Helen Berman, and Eric Westhof. Tools for the automatic identification and classification of rna base pairs. *Nucleic acids research*, 31(13):3450–3460, 2003.

[12] Bernhard C Thiel, Irene K Beckmann, Peter Kerpedjiev, and Ivo L Hofacker. 3d based on 2d: Calculating helix angles and stacking patterns using forgi 2.0, an rna python library centered on secondary structure elements. *F1000Research*, 8:ISCB–Comm, 2019.

[13] Wolfgang Kabsch and Christian Sander. Dictionary of protein secondary structure: Pattern recognition of hydrogen-bonded and geometrical features. *Biopolymers*, 22(12):2577–2637, December 1983.

[14] John Jumper, Richard Evans, Alexander Pritzel, Tim Green, Michael Figurnov, Olaf Ronneberger, Kathryn Tunyasuvunakool, Russ Bates, Augustin Žídek, Anna Potapenko, et al. Highly accurate protein structure prediction with alphafold. *nature*, 596(7873):583–589, 2021.

[15] Milot Mirdita, Konstantin Schütze, Yoshitaka Moriwaki, Lim Heo, Sergey Ovchinnikov, and Martin Steinegger. Colabfold: making protein folding accessible to all. *Nature Methods*, 19(6):679–682, May 2022.

[16] He Zhang, Liang Zhang, David H Mathews, and Liang Huang. Linearpartition: linear-time approximation of rna folding partition function and base-pairing probabilities. *Bioinformatics*, 36(Supplement_1):i258–i267, 2020.

[17] Yang Zhang and Jeffrey Skolnick. Scoring function for automated assessment of protein structure template quality. *Proteins: Structure, Function, and Bioinformatics*, 57(4):702–710, 2004.

[18] Valerio Mariani, Marco Biasini, Alessandro Barbato, and Torsten Schwede. lddt: a local superposition-free score for comparing protein structures and models using distance difference tests. *Bioinformatics*, 29(21):2722–2728, 2013.

[19] Adam Zemla. Lga: a method for finding 3d similarities in protein structures. *Nucleic acids research*, 31(13):3370–3374, 2003.

[20] Adam Zemla, Česlovas Venclovas, John Moult, and Krzysztof Fidelis. Processing and evaluation of predictions in casp4, 2001.

[21] Gabriel Studer, Gerardo Tauriello, and Torsten Schwede. Assessment of the assessment—all about complexes. *Proteins: Structure, Function, and Bioinformatics*, 91(12):1850–1860, 2023.

[22] Sheng-You Huang and Xiaoqin Zou. A knowledge-based scoring function for protein-rna interactions derived from a statistical mechanics-based iterative method. *Nucleic acids research*, 42(7):e55–e55, 2014.

[23] Sheng-You Huang and Xiaoqin Zou. A nonredundant structure dataset for benchmarking protein-rna computational docking. *Journal of computational chemistry*, 34(4):311–318, 2013.

[24] Chengwei Zeng, Yiren Jian, Soroush Vosoughi, Chen Zeng, and Yunjie Zhao. Evaluating native-like structures of rna-protein complexes through the deep learning method. *Nature Communications*, 14(1):1060, 2023.

[25] Aleix Lafita, Spencer Bliven, Andriy Kryshtafovych, Martino Bertoni, Bohdan Monastyrskyy, Jose M Duarte, Torsten Schwede, and Guido Capitani. Assessment of protein assembly prediction in casp12. *Proteins: Structure, Function, and Bioinformatics*, 86:247–256, 2018.

[26] Kliment Olechnovič, Rita Banciul, Justas Dapkūnas, and Česlovas Venclovas. Ftdmp: A framework for protein–protein, protein–dna, and protein–rna docking and scoring. *Proteins: Structure, Function, and Bioinformatics*, 2025.

[27] Irina Tuszynska and Janusz M Bujnicki. Dars-rnp and quasi-rnp: new statistical potentials for protein-rna docking. *BMC bioinformatics*, 12:1–16, 2011.

[28] Martin Steinegger and Johannes Söding. Mmseqs2 enables sensitive protein sequence searching for the analysis of massive data sets. *Nature biotechnology*, 35(11):1026–1028, 2017.

[29] Nancy Ontiveros-Palacios, Emma Cooke, Eric P Nawrocki, Sandra Triebel, Manja Marz, Elena Rivas, Sam Griffiths-Jones, Anton I Petrov, Alex Bateman, and Blake Sweeney. Rfam 15: Rna families database in 2025. *Nucleic acids research*, 53(D1):D258–D267, 2025.

[30] Josh Abramson, Jonas Adler, Jack Dunger, Richard Evans, Tim Green, Alexander Pritzel, Olaf Ronneberger, Lindsay Willmore, Andrew J Ballard, Joshua Bambrick, et al. Accurate structure prediction of biomolecular interactions with alphafold 3. *Nature*, 630(8016):493–500, 2024.
